# Analysis of trio test in neurodevelopmental disorders

**DOI:** 10.3389/fped.2022.1073083

**Published:** 2022-12-23

**Authors:** Se Hee Kim, Soon Sung Kwon, Joon Soo Lee, Heung Dong Kim, Seung-Tae Lee, Jong Rak Choi, Saeam Shin, Hoon-Chul Kang

**Affiliations:** ^1^ Department of Pediatrics, Yonsei University College of Medicine, Seoul, South Korea; ^2^ Department of Laboratory Medicine, Yonsei University College of Medicine, Seoul, South Korea

**Keywords:** neurodevelopmental disorder, genetic diagnosis, next-generation sequencing, trio test, *de novo* variant

## Abstract

**Background:**

Trio test has been widely used for diagnosis of various hereditary disorders. We aimed to investigate the contribution of trio test in genetically diagnosing neurodevelopmental disorders (NDD).

**Methods:**

We retrospectively reviewed 2,059 NDD cases with genetic test results. The trio test was conducted in 563 cases. Clinical usefulness, optimal timing, and methods for the trio test were reviewed.

**Results:**

Pathogenic or likely pathogenic variants were detected in 112 of 563 (19.9%) patients who underwent the trio test. With trio test results, the overall diagnostic yield increased by 5.4% (112/2,059). Of 165 *de novo* variants detected, 149 were pathogenic and we detected 85 novel pathogenic variants. Pathogenic, *de novo* variants were frequently detected in *CDKL5*, *ATP1A3*, and *STXBP1*.

**Conclusion:**

The trio test is an efficient method for genetically diagnosing NDD. We identified specific situations where a certain trio test is more appropriate, thereby providing a guide for clinicians when confronted with variants of unknown significance of specific genes.

## Introduction

Neurodevelopmental disorders (NDD) are a group of disorders caused by abnormal brain development, which result in impaired brain functions. NDD include a heterogeneous group of diseases such as epilepsy, intellectual disability, autism, developmental delay, and other various neuropsychiatric diseases (1, 2). These disorders may cause serious life-long functional deficits. In the United States, a nationwide surveillance during 2014–2016 showed the following prevalence of NDD: 1.1%–1.3% for intellectual disability, 2.2%–2.7% for autism spectrum disorder, and 3.6%–4.6% for developmental delay (3). NDD may be caused by multiple factors affecting normal brain development, such as genetics, environment, toxins, infections, and injuries. Identifying the underlying genetic causes of NDD in patients is important for proper patient management, genetic counseling, and prediction of prognosis, as well as for understanding the pathophysiology of NDD.

To date, a large number of genes involved in the occurrence of NDD have been identified. However, there are still several patients with NDD for whom the causative variants have not yet been identified and only variants of unknown significance (VOUS) have been detected from several known or probable NDD-related genes. These variants cannot be determined as either pathogenic or benign owing to the lack of evidence. In practice, the easiest way of accessing the pathogenicity of detected variants is through a parental test with a proband test, also known as the trio test. When a certain variant is identified as a *de novo* variant, it provides powerful evidence that supports the pathogenicity of the variant.

In this study, we reviewed results of trio tests for suspected variants and identified NDD-causing pathogenic variants. The results of the trio tests of 563 patients with NDD are described, in which we detected 149 pathogenic *de novo* variants with several novel variants of various NDD-related genes. The results suggest the clinical usefulness of the trio test for the diagnosis of NDD with genetic causes. We also investigated whether there are differences in diagnostic yield according to the timing of trio test and trio test methods.

## Materials and methods

### Participants and trio testing

A total of 2,059 patients were referred for genetic diagnosis of various NDD at a tertiary hospital in Seoul, Korea, from 2016 to 2021. The clinical history and genetic test results of the patients were retrospectively reviewed for this study. Among them, individuals with trio test results were further investigated. A trio test is usually ordered by the primary care physician after a proband study if candidate genes or VOUS were found. However, sometimes a trio test is performed earlier for some patients if the family wanted to receive quick test result for reasons such as genetic counseling. Sometimes, the physician would not offer a trio test if no good gene candidate was identified in the first test report. In some cases, the parents refused to take the offered trio test because of the cost.

The types of trio tests included NGS-based trio test and Sanger sequencing (for each variant detected). The cases were discussed at a multidisciplinary meeting held with neurologists, geneticists, and bioinformaticians. Additional evaluations or the type of trio test were usually determined after the meeting.

Clinical variables, such as age, sex, and diagnosis, were collected from the electronic medical record review.

This study was conducted following the standards of the Declaration of Helsinki and was approved by the Institutional Review Board (IRB) of the Yonsei College of Medicine (4-2022-0327). The IRB also approved a waiver of informed consent because all patients information were de-identified and this study was conducted as a retrospective study.

### Sequencing

Genomic DNA was extracted from the peripheral blood of patients and their parents. For an NGS-based trio test, libraries were prepared, and targets were captured using various custom panels for candidate genes (gene panels and genes included in each panel are listed in Supplementary Table S1). The prepared and pooled libraries were sequenced using NextSeq 550Dx System (Illumina, San Diego, CA, United States). The sequencing data were analyzed using the custom bioinformatics pipeline as previously described (4, 5). For the test, the parents underwent the same NGS panel testing that the patient received.

For a Sanger sequencing trio test, gene regions that included the target variants were amplified by target-specific primers and further sequenced using a 3,730 DNA Analyzer with BigDye Terminator v3.1 Cycle Sequencing Kit (Applied Biosystems, Foster City, CA, USA).

### Variant interpretation

Variants were classified based on the recommendation of the American College of Medical Genetics and Genomics (6). Among the variants identified from the bioinformatics analyses, the benign and likely benign variants were sorted based on the location of the variant and population frequency reported from the 1,000 Genomes project, Genome Aggregation Database, Exome Sequencing Project, and Korean Reference Genome Database. Variants that were not classified as benign or likely benign were further reviewed by geneticists to determine the pathogenicity of the variant. Literature and database reviews and in silico analyses were performed by geneticists. Genotype–phenotype correlation of the variants was additionally performed by pediatric neurologists and geneticists. The interpretations of the variants were reviewed and reclassified based on the result of the trio test.

### Descriptive statistics

The time for the trio test was presented as median, minimum, maximum, and interquartile ranges (IQR). Descriptive statistics were calculated using Microsoft Excel (Microsoft, Redmond, WA, USA).

## Results

### Patient demographics

**Figure 1:**
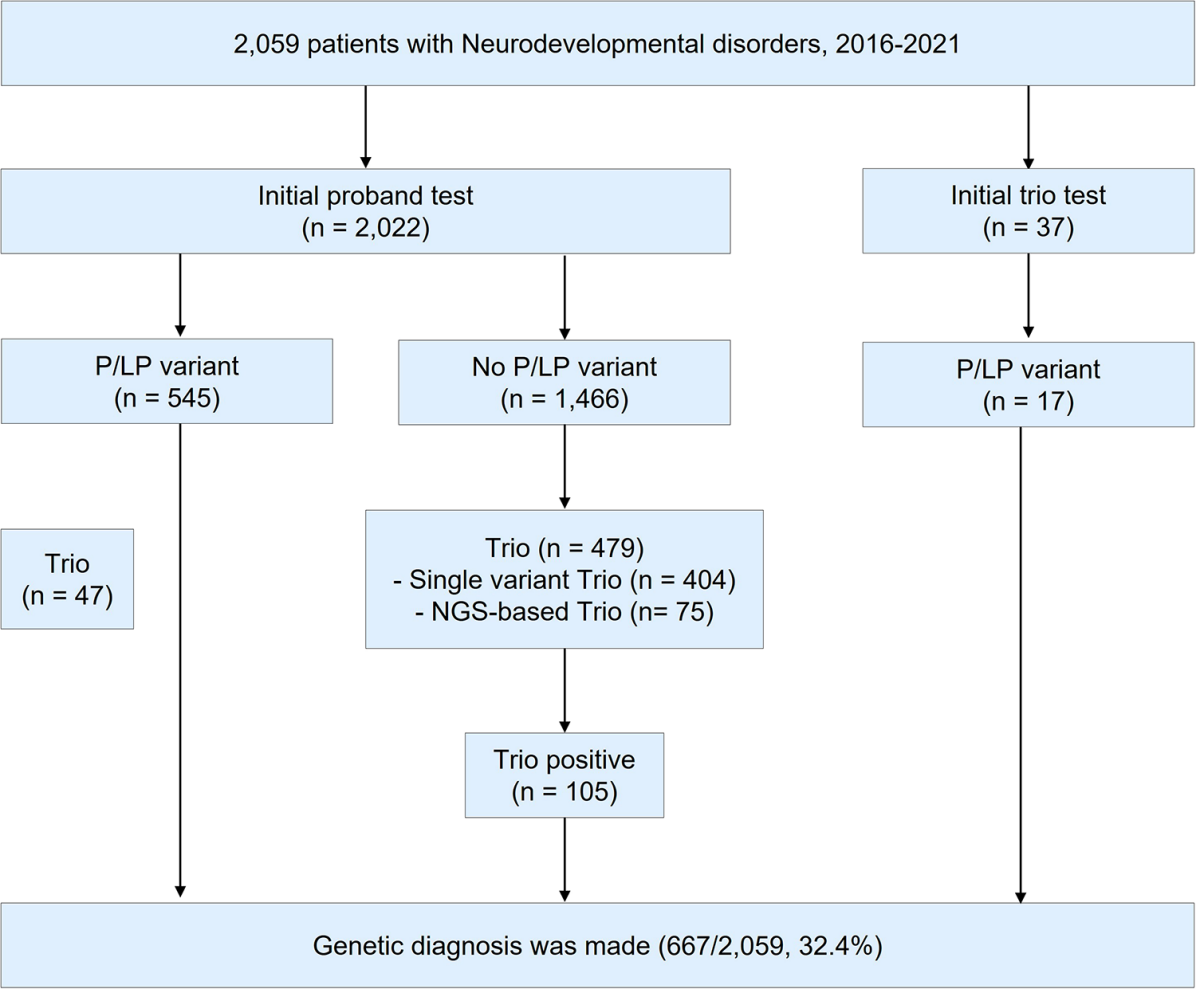
Study design. During 2016–2021, a total of 2,059 patients were tested for the diagnosis of neurodevelopmental disorders (NDD). P/LP variant; pathogenic/likely pathogenic variant. NGS; next-generation sequencing.

During 2016–2021, a total of 2,059 patients with NDD were requested for an NGS test to determine the causal pathogenic variants (Figure 1). In this study, 1,212 patients underwent clinical exome, 755 patients underwent epilepsy-targeted gene panel, and 92 patients underwent malformation of cortical development-targeted gene panel testing. Of the 2,059 patients, 1,184 were male (57.5%), and the median age was 54.8 months (0–794.8).

Of the 2,059 patients, trio test was performed for 563 patients to improve interpretation. Of the 563 patients, 332 were male (59.0%), and the median age was 44.4 months (0.3–321.6). A total of 410 out of the 563 patients (72.8%) were patients with NDD with epilepsy. Clinical exome was the most frequently used for diagnosis (342/563, 60.7%), followed by an epilepsy panel (206/563, 36.6%) and malformation of cortical development panel (15/563, 2.7%). A single gene trio test was performed using Sanger sequencing in 442 cases (78.5%), while an NGS-based trio was performed in 121 cases (21.5%). Detailed demographics of the patients are presented in Supplementary Table S2.

### Trio test for the diagnosis of NDD

The overall diagnostic yield of NGS for NDD was 32.4% (667/2,059). Among these, 112 cases were confirmed to have pathogenic or likely pathogenic variants by trio test, which improved the diagnostic yield by 5.4% (112/2,059). The median time for the trio test performed from the initial test report to the final report was 2.1 months (0–48.9, IQR: 1.3–3.5). The diagnostic yield, number of cases with trio tests, number of cases solved by trio tests, and *de novo* variants of each targeted gene panel are presented in Table 1. Among those 563 patients with trio test, trio test was conducted at the initial molecular diagnosis in 37 patients (Supplementary Table S3).

**Table 1:** Statistics of targeted gene panels.

	CE (*n* = 1,212)	Epilepsy (*n* = 755)	MCD (*n* = 92)
Pathogenic/Likely pathogenic variant	426 (35.1)	205 (27.2)	36 (39.1)
Trio tested	342 (28.2)	206 (27.3)	15 (16.3)
Cases solved by trio test	72 (5.9)	36 (4.8)	4 (4.3)
*De novo* variants	112 (9.2)	47 (6.2)	6 (6.5)

CES, clinical exome; MCD, malformation of cortical development.

### Cases with *de novo* variants

The trio test detected 165 *de novo* variants from 159 patients, of which 149 variants proved to be pathogenic or likely pathogenic. Missense variants were the most frequent type (99/149, 66.4%), followed by nonsense (11/149, 7.4%), splicing site (10/149, 6.7%), gene or chromosomal deletion (10/149, 6.7%), duplication (5/149, 3.4%), and indel (4/149, 2.7%) variants. Among the detected *de novo* variants, 85 variants were novel, which were listed in Table 2. The novel variants were frequently identified from the following genes: *STXBP1* (7 cases), *SCN2A* (7 cases), *CDKL5* (4 cases), *GABRA1* (3 cases), *DNM1* (3 cases), and *SCN1A* (3 cases).

**Table 2:** Novel *de novo* pathogenic/likely pathogenic variants detected in patients with NDD.

Trio#	NDD with epilepsy	Gene	Transcript	Nucleotide	Amino acid	Interpretation
14	No	*ADCY5*	NM_183357.2	c.1235G > T	p.Arg412Leu	LP
40	No	*ARID1B*	NM_020732.3	c.3051delG	p.Met1017IlefsTer113	P
48	No	*ATP1A3*	NM_152296.4	c.2528C > A	*p*.Ala843Asp	LP
57	Yes	*CACNA1A*	NM_001127221.1	c.4178T > C	p.Val1393Ala	LP
61	No	*CACNA1C*	NM_000719.6	c.770T > C	p.Val257Ala	LP
67	Yes	*CACNA1H*	NM_021098.2	c.1654C > T	p.Arg552Ter	LP
76	No	*CDKL5*	NM_003159.2	c.2684C > T	p.Pro895Leu	LP
78	Yes	*CDKL5*	NM_003159.2	c.80T > C	p.Val27Ala	LP
79	Yes	*CDKL5*	NM_003159.2	c.595T > C	p.Cys199Arg	LP
80	Yes	*CDKL5*	NM_003159.2	c.146-1G > T		P
82	Yes	*CHD2*	NM_001271.3	c.4137 + 3A > G		LP
83	Yes	*CHD2*	NM_001271.3	c.2605_2606delinsTT	p.Ala869Phe	LP
103	Yes	*COL4A1*	NM_001845.5	c.3922G > A	p.Gly1308Arg	LP
124	Yes	*DNM1*	NM_004408.2	c.1943T > C	p.Met648Arg	LP
127	Yes	*DNM1*	NM_001005336.1	c.415_423del	p.Gly139_Thr141del	LP
128	Yes	*DNM1*	NM_001005336.1	c.632A > T	p.Asp211Val	LP
129	Yes	*DNM1l*	NM_012062.4	c.1247T > C	p.Leu416Pro	LP
134	No	*DYNC1H1*	NM_001376.4	c.12419G > A	p.Arg4140His	LP
137	Yes	*EEF1A2*	NM_001958.3	c.43C > A	p.His15Asn	LP
142	No	*FOXP1*	NM_032682.5	c.573dup	p.Gln192ThrfsTer103	P
144	No	*FOXP1*	NM_032682.5	c.58°C > T	p.Gln194Ter	P
148	Yes	*GABRA1*	NM_000806.5	c.134T > C	p.Ile45Thr	LP
149	Yes	*GABRA1*	NM_000806.5	c.839C > T	p.Pro280Leu	LP
150	Yes	*GABRA1*	NM_000806.5	c.84G > T	p.Gln28His	LP
152	Yes	*GABRB2*	NM_021911.2	c.929T > C	p.Met310Thr	LP
156	Yes	*GABRG2*	NM_198903.2	c.631 + 4A > G		LP
169	No	*GRIN2B*	NM_000834.3	c.1237G > A	p.Glu413Lys	LP
175	Yes	*GRIN2D*	NM_000836.2	c.233°C > T	p.Thr777Ile	LP
179	Yes	*HCN1*	NM_021072.3	c.794T > A	p.Leu265His	LP
180	Yes	*HCN1*	NM_021072.3	c.535A > T	p.Asn179Tyr	LP
184	No	*HNRNPK*	NM_002140.3	c.1192-7_1192-3del		P
186	Yes	*HNRNPU*	NM_031844.2	c.61A > G	p.Lys21Glu	LP
194	Yes	*IQSEC2*	NM_001111125.2	c.2295C > G	p.Asn765Lys	LP
196	Yes	*IQSEC2*	NM_001111125.2	c.3016-1G > T		P
204	Yes	*KAT6A*	NM_006766.3	c.4667T > C	p.Ile1556Thr	LP
207	Yes	*KCNA2*	NM_004974.3	c.785C > T	p.Ala262Val	LP
208	Yes	*KCNA2*	NM_004974.3	c.1130A > G	p.Tyr377Cys	LP
214	Yes	*KCNB1*	NM_004975.3	c.1237G > A	p.Val413Ile	LP
219	Yes	*KCNB1*	NM_004975.2	c.1106G > T	p.Trp369Leu	LP
230	Yes	*KCNQ2*	NM_172107.2	c.2331dup	p.Glu778ArgfsTer87	P
253	No	*KIF1A*	NM_001244008.1	c.806C > A	p.Ala269Asp	LP
258	No	*MBD5*	NM_018328.4	c.1756G > A	p.Ala586Thr	LP
260	No	*MECP2*	NM_004992.3	c.901C > G	p.Leu301Val	LP
287	No	*OTUD7A*	NM_130901.2	c.1230G > A	p.Trp410Ter	P
291	Yes	*PAFAH1B1*	NM_000430.3	c.485G > A	p.Gly162Asp	LP
299	Yes	*PCDH19*	NM_001184880.1	c.1728C > G	p.Tyr576Ter	P
324	No	*PTEN*	NM_000314.6	c.374A > G	p.Lys125Glu	LP
326	No	*PURA*	NM_005859.4	c.227A > T	p.Asp76Val	LP
341	Yes	*SCN1A*	NM_006920.4	c.1006T > C	p.Cys336Arg	LP
351	Yes	*SCN1A*	NM_006920.4	c.2638G > A	p.Gly880Arg	LP
358	Yes	*SCN1A*	NM_006920.4	c.3970-4T > G		LP
361	No	*SCN2A*	NM_001040142.1	c.3676-7C > G		LP
364	No	*SCN2A*	NM_001040142.1	c.5636T > C	p.Met1879Thr	LP
367	No	*SCN2A*	NM_001040142.1	c.183_184insA	p.Leu62ThrfsTer27	P
368	Yes	*SCN2A*	NM_001040142.1	c.4499C > T	p.Ala1500Val	LP
369	Yes	*SCN2A*	NM_001040142.1	c.4426T > A	p.Phe1476Ile	LP
370	Yes	*SCN2A*	NM_001040142.1	c.5636T > C	p.Met1879Thr	LP
371	Yes	*SCN2A*	NM_001040142.1	c.819C > A	p.Asn273Lys	LP
375	Yes	*SCN2A*	NM_001040142.1	c.4622T > A	p.Ile1541Asn	LP
381	Yes	*SCN8A*	NM_014191.3	c.2934C > A	p.Ser978Arg	LP
386	Yes	*SCN8A*	NM_014191.3	c.778T > G	p.Phe260Val	LP
387	Yes	*SCN8A*	NM_014191.3	c.2934C > A	p.Ser978Arg	LP
389	Yes	*SCN8A*	NM_014191.3	c.4871T > G	p.Ile1624Ser	LP
391	Yes	*SCN8A*	NM_014191.3	c.4475T > C	p.Met1492Thr	LP
407	Yes	*SETD1B*	NM_001353345.2	c.5860T > C	p.Cys1911Arg	LP
410	No	*SHANK2*	NM_133266.3	c.3412_3413del	p.Leu1138ValfsTer16	LP
413	No	*SHANK3*	NM_001372044.2	c.4908C > G	p.Tyr1561Ter	P
420	Yes	*SIN3A*	NM_001145358.1	c.366 + 5T > C		LP
423	Yes	*SLC1A2*	NM_004171.3	c.872G > T	p.Gly291Val	LP
429	Yes	*SLC6A1*	NM_003042.3	c.896G > T	p.Gly299Val	LP
435	Yes	*SON*	NM_032195.2	c.5455_5456dup	p.Asp1819GlufsTer5	LP
444	Yes	*STX1B*	NM_052874.4	c.655_660dup	p.Ala219_Met220dup	LP
446	Yes	*STXBP1*	NM_003165.3	c.701A > G	p.Asp234Gly	LP
447	Yes	*STXBP1*	NM_003165.3	c.1657G > C	p.Ala553Pro	LP
449	No	*STXBP1*	NM_003165.3	c.328T > C	p.Cys110Arg	LP
450	No	*STXBP1*	NM_003165.3	c.620A > G	p.Asp207Gly	LP
452	Yes	*STXBP1*	NM_003165.3	c.748C > G	p.Gln250Glu	LP
454	Yes	*STXBP1*	NM_003165.3	c.1030-2A > G		P
455	Yes	*STXBP1*	NM_003165.3	c.1003C > T	p.Pro335Ser	LP
483	Yes	*TSC2*	NM_000548.3	c.1358_1361 + 14del		P
484	Yes	*TUBA1A*	NM_006009.3	c.626T > C	p.Ile209Thr	LP
485	No	*TUBA1A*	NM_006009.3	c.615C > A	p.Asp205Glu	LP
489	Yes	*UBE3A*	NM_130838.1	c.2398_2401dup	p.Lys801IlefsTer23	P
495	Yes	*ZBTB18*	NM_205768.2	c.1478A > G	p.His493Arg	LP
497	No	*ZNF462*	NM_021224.5	c.5941C > T	p.Arg1981Ter	P

LP, likely pathogenic; P, pathogenic.

A total of 16 *de novo* variants remained as VOUS after the trio test (Supplementary Table S4). Most of these variants were not reported in the general population and related patients, and the phenotypic correlations were weak. The variants could not be classified as pathogenic or likely pathogenic although they were confirmed to occur *de novo*.

The target variants and genes requested for trio testing are listed in Table 3. The most frequently requested gene was *SCN1A*, followed by *SCN2A*, *ADGRV1*, *SCN8A*, *TSC2*, and *CHD2*. Notably, all the variants of three genes—*CDKL5* (6/6, 100%), *ATP1A3* (3/3, 100%), and *STXBP1* (9/11, 81.8%)—were confirmed as *de novo* and causative. By contrast, the variants of the following genes were rarely confirmed to be *de novo*: *ADGRV1* (1/14), *SCN9A* (0/11) and *SETBP1* (0/10). For 37 genes, no *de novo* variants were detected.

**Table 3:** Trio-tested genes and the number of *de novo* variants detected.

Gene	Trio-tested	*De novo*	%
*SCN1A*	21	6	28.6
*SCN2A*	19	11	57.9
*ADGRV1*	14	1	7.1
*SCN8A*	13	9	69.2
*TSC2*	12	2	16.7
*CHD2*	12	5	41.7
*STXBP1*	12	10	83.3
*PCDH19*	10	2	20.0
*SHANK3*	9	1	11.1
*SPTAN1*	9	1	11.1
*ARID1B*	9	3	33.3
*CACNA1A*	8	1	12.5
*KCNQ2*	7	2	28.6
*GABRA1*	7	4	57.1
*KCNB1*	6	1	16.7
*DNM1*	6	3	50.0
*CDKL5*	6	6	100.0
*MED13L*	5	1	20.0
*COL4A1*	4	1	25.0
*FOXP1*	4	1	25.0
*KIF1A*	4	1	25.0
*SMARCA2*	4	1	25.0
*TBC1D24*	4	1	25.0
*DEAF1*	4	2	50.0
*HCN1*	4	2	50.0
*SLC2A1*	4	2	50.0
*ATP1A3*	4	4	100.0
*CACNA1H*	3	1	33.3
*GABRB3*	3	1	33.3
*HNRNPU*	3	1	33.3
*PRRT2*	3	1	33.3
*TSC1*	3	1	33.3
*EEF1A2*	3	2	66.7
*MECP2*	3	2	66.7

Genes trio-tested three or more times were listed in this table. Genes without *de novo* variants were not listed in the table. Gene (number of trio test): *SCN9A* (11), *SETBP1* (10), *KCNT1* (9), *KCNMA1* (7), *POLG* (7), *SCN3A* (7), *GRIN2A* (6), *GRIN2B* (6), *KCNQ3* (6), *ALDH7A1* (5), *CHD8* (5), *DEPDC5* (5), *KANSL1* (5), *KCNA2* (5), *SCN1B* (5), *SZT2* (5), *WWOX* (5), *ARX* (4), *CHD7* (4), *CHRNA4* (4), *HUWE1* (4), *KAT6A* (4), *PHIP* (4), *SIK1* (4), *SLC6A1* (4), *ALG13* (3), *BRAT1* (3), *CACNB4* (3), *CASR* (3), *CHRNB2* (3), *KMT2D* (3), *MYT1l* (3), *NDUFS2* (3), *PACS1* (3), *SETD2* (3), and *ZEB2* (3).

## Discussion

Recent progress in NGS tests has improved the cost-effectiveness of the NGS-based trio tests, thereby encouraging its use. NGS-based trio tests can determine novel candidate genes and variants and find causal pathogenic variants, which can be overlooked during variant interpretation. Exome-trio has recently been widely used, and studies have reported its clinical usefulness for the diagnosis of NDD (7–9). In these studies, various number of patients with NDD were trio-tested (54–244 patients). Diagnostic yields ranged from 25.2% to 57.4%. In this study, the trio test detected pathogenic causal variants in 22.3% (112/503) of the cases, which might not have been interpreted as pathogenic without the trio test. This resulted in an increase in the overall diagnostic yield of NDD from 27.0% to 32.4%, which encouraged the use of trio tests for the genetic diagnosis of NDD. A trio test is a powerful tool that offers a high diagnostic yield, while proband-only clinical exome sequencing provides a cost-effective approach to NDD (5).

*De novo* variants rarely occur in an individual, and the occurrence of *de novo* variants in the coding region of a certain gene is even rarer (10, 11). For this reason, a *de novo* variant in a patient with a family history consistent with *de novo* inheritance is highly suspected as the causal pathogenic variant for NDD. Guidelines for the interpretation of sequence variants regarded *de novo* occurrence of a variant as strong evidence for pathogenicity (6). The results also supported the high correlation between *de novo* occurrence of the variants and their pathogenicity, with 86.1% of the trio-tested pathogenic variants (149/173) occurring *de novo*. This high rate of *de novo* variants was consistent with previous studies, which reported the rate of *de novo* variants 80% and 61.5%, respectively (8, 9).

To have a high detection yield with a single gene trio, variants to be trio-tested need to be selected carefully. First, the genotype-phenotype correlation between the gene and patient should exist. Second, variants that are not detected in a normal healthy population were selected. Third, the previous test results were reviewed and checked if the target gene has been frequently reported with *de novo* variants. These genes included *CDKL5*, *ATP1A3*, and *STXBP1*. These genes were already known for their high frequency of *de novo* variants (12–17). On the contrary, there are situations where the trio test is less likely to be helpful when genes with low rates of *de novo* variants are detected. There are genes in which pathogenic variants were rarely found. In this study, *de novo* variants were rarely detected in *ADGRV1*, *SCN9A*, *SETBP1*, and *KCNT1*. Non-pathogenic, inherited VOUS are frequently reported in these genes, probably owing to their relatively large gene size. Calculated scoring systems for the prediction of pathogenicity, such as the *Z*-score and MutScore, can aid in determining the need for trio testing (18, 19).

In this study, most of the trio tests were performed after the initial NGS test results were determined negative. Only 37 cases underwent an initial trio. For the cases with initial trio, the diagnostic yield was high, reaching approximately 50% (17/37), and the diagnosis could be made immediately. By contrast, a retrospective review of these cases also demonstrated that some of those variants (10/17, 58.8%) could have been classified as pathogenic without the trio test. In addition, there was no difference in the diagnostic rate of cases solved by the trio test between the initial trio and delayed trio tests (18.9% vs. 20.0%). The optimal timing of the trio test has not yet been established. The clinical setting, phenotypes, socio-economic status of parents, and experience of the clinicians would have to be considered when pediatric neurologists and geneticists decide when to perform the trio test.

Trio test identified cases with parental mosaicism. Parental mosaicism of *SLC2A1* has been reported (20, 21), and the level of mosaicism appears to be related to symptoms, which is consistent with the results of this study. Parental mosaicisms of *SPTAN1* and *RORA* have not been reported yet to the best of our knowledge. Parental pathogenic mosaic variants should be addressed considering that they increase the risk of disease recurrence in future offsprings (22, 23). A recent study reported the contribution of low-level parental mosaic disease-causing variants to NDD (24). In cases of parental testing conducted by Sanger sequencing or low-depth NGS tests, low-level parental mosaicisms may be missed during diagnosis. A sensitive NGS-based trio test along with proper noting of family history can identify such cases.

In this study, we reported 16 *de novo* VOUS. In these cases, variants could not be classified as pathogenic owing to the lack of evidence. For example, a frameshift variant of *RBM12* was detected in a patient with epilepsy and mildly delayed development. The correlation between pediatric NDD and *RBM12* has not been reported yet, which led to this variant being classified as VOUS. However, one report demonstrated that a truncating variant of *RBM12* is associated with psychosis (25), which remains to be a possible relation between the *RBM12* variant and the phenotype of the patient. A missense *de novo* variant of *CUX1* was detected in a patient with epilepsy, autism, and intellectual disability. Although the *de novo* variant of *CUX1* had been reported in patients with delayed development (26), all the reported cases were of truncating variants. Thus, the variant was reported as VOUS. There were *de novo* variants that lacked phenotypic consistency; a splicing site variant of *ATP8A1* was detected in a patient with autism spectrum disorder and delayed development who did not have any symptoms and signs related to cholestasis. A splicing site variant of *COPA*, a gene-related to autoimmune diseases (27), was identified as a *de novo* occurrence in a patient with delayed development and epilepsy without other abnormalities. Pathogenic variants of *DNM2* were known to cause myopathy and Charcot–Marie–Tooth disease (28, 29). Additionally, a frameshift variant occurred *de novo* in a patient with epilepsy who did not demonstrate the phenotype related to DNM2-related diseases. Detected *de novo* variants of other genes (*CTC1*, *TDRD7*, *RANBP17*, *RABL6*, *DLX6*, *LDHA*, *VPS13A*, *LAMA3*, *GAL3ST2*, and *GATA3*) were classified as VOUS, considering these genes lack disorder-related reports. Although these kinds of variants should be interpreted with caution, they may be novel variants of NDD, which may be identified as causal variants in future re-analyses.

Advances in the genetic diagnostic methods for NDD have now led to the identification of numerous variants, which requires development of methods to interpret their clinical meaning. This study demonstrated the results of the trio test with several novel pathogenic variants causing NDD. The data demonstrated that the trio test is a powerful tool to determine causal pathogenic variants among the variants from patients with NDD. The study also describes specific situations where the trio test is more or less useful, which can provide clinicians with a guide when confronted with VOUS of specific genes.

## Data Availability

The data presented in the study are deposited in the ClinVar repository, accession number SCV002761259 to SCV002761341.
