# Supplementary material for: Analysis of trio test in neurodevelopmental disorders
Source: Front Pediatr. 2022 Dec 23;10:1073083. doi: 10.3389/fped.2022.1073083 (PMC9816327; doi:10.3389/fped.2022.1073083)
Supplement: Supplementary file 1 [file Datasheet1.pdf]

Supplementary Material

Trio#	Gene	Transcript	Nucleotide	AminoAcid	Diagnosis	Interpretation	Mosaicism
92	*SLC2A1*	NM_006516.2	c.1156_1157dup	p.Pro387SerfsTer122	Ataxia<br>Dystonia	Likely pathogenic	Paternal mosaicism
220	*SPTAN1*	NM_001130438.2	c.2482G>A	p.Ala828Thr	Delayed development<br>Muscle disorder	Likely pathogenic	Maternal mosaicism
411	*RORA*	NM_134261.2	c.1336C>T	p.Gln446Ter	Delayed development<br>Epilepsy	Likely pathogenic	Maternal mosaicism

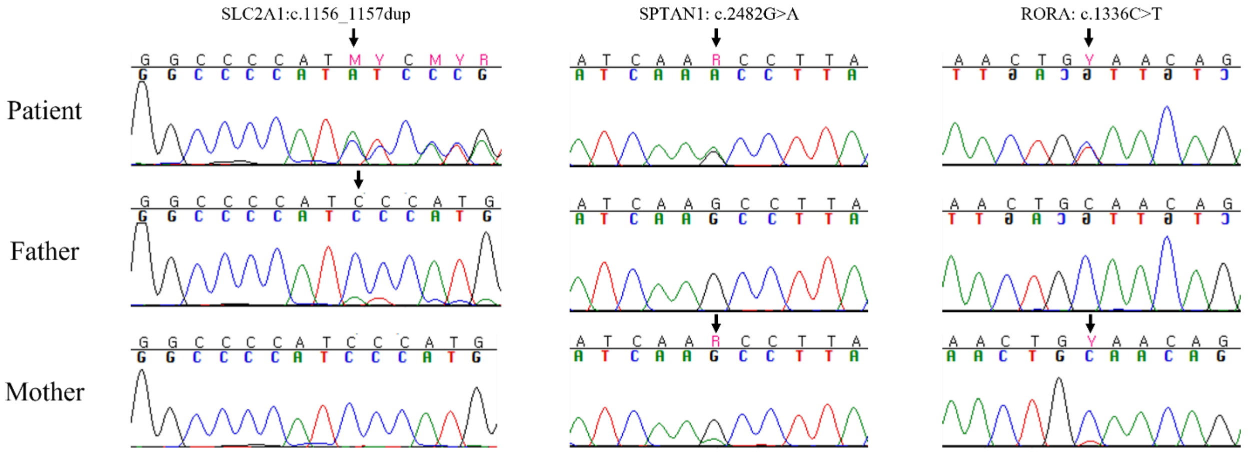

Supplementary Figure 1. Parental mosaicism cases. Pathogenic variants detected from the probands were described in the upper panel. Sanger sequencing data of each patient and parents demonstrated parental mosaicism of detected variants (variants of interests are indicated by black arrows)

Supplementary Table 1. Gene lists of next-generation sequencing gene panels.

Panel (No. of included genes)	Genes
Epilepsy (n=218)	*AARS, ABAT, ACADL, ACADM, ACADS, ACY1, ADGRV1, ADSL, ALAD, ALAS2, ALDH4A1, ALDH7A1, ALG13, ALPL, AMT, ARHGEF15, ARHGEF9, ARX, ASNS, ASPM, ATP13A2, ATP6AP2, BRAT1, BTD, CACNA1A, CACNB4, CASK, CASR, CBS, CDKL5, CHD2, CHRNA2, CHRNA4, CHRNA7, CHRNB2, CLCN4, CLN3, CLN5, CLN6, CLN8, CNTNAP2, COL4A1, CPOX, CPT1A, CPT1B, CPT2, CSTB, CTSD, CTSF, DNAJC5, DNM1, DOCK7, DYRK1A, EEF1A2, EPM2A, FARS2, FECH, FOLR1, FOXG1, GABBR2, GABRA1, GABRB3, GABRG2, GAMT, GATM, GCSH, GLDC, GNAO1, GOSR2, GRIN1, GRIN2A, GRIN2B, GRN, HADH, HADHA, HCN1, HCN4, HFE, HLCS, HMBS, HNRNPU, IQSEC2, KANSL1, KCNA1, KCNA2, KCNB1, KCNC1, KCNH5, KCNJ10, KCNJ11, KCNMA1, KCNQ2, KCNQ3, KCNT1, KCTD7, KPNA7, LGI1, LIAS, MAGI2, MBD5, MECP2, MEF2C, MFSD8, MMADHC, MTHFR, MTR, MTRR, NECAP1, NHLRC1, NRXN1, OPHN1, PAH, PC, PCDH19, PHGDH, PIGA, PIGQ, PLCB1, PNKP, PNPO, POLG, PPOX, PPT1, PRICKLE1, PRICKLE2, PRODH, PRRT2, PURA, QARS, SCARB2, SCN1A, SCN1B, SCN2A, SCN3A, SCN8A, SCN9A, SETBP1, SIK1, SLC13A5, SLC19A3, SLC22A5, SLC25A20, SLC25A22, SLC25A29, SLC2A1, SLC46A1, SLC6A1, SLC6A8, SLC9A6, SMARCA2, SPTAN1, SRPX2, ST3GAL3, ST3GAL5, STX1B, STXBP1, SYN1, SYNGAP1, SZT2, TBC1D24, TBL1XR1, TCF4, TNK2, TPP1, TSEN54, UBE2A, UBE3A, UROD, UROS, WDR62, WWOX, ZEB2, ADRA2B, AP3B2, ARV1, ASAH1, ATP1A2, CACNA1H, CACNA2D2, CAD, CERS1, CNTN2, CPA6, DENND5A, DEPDC5, EFHC1, FGF12, FRRS1L, GABRA6, GABRB1, GABRB2, GABRD, GAL, GRIN2D, GUF1, HCN2, IER3IP1, ITPA, KCND2, KCND3, KCNV2, LMNB2, NPRL2, NPRL3, PIGP, PPP3CA, PRDM8, RELN, SHANK3, SLC12A5, SLC1A2, SLC25A12, SNIP1, STRADA, SYNJ1, UBA5, YWHAG, CLCN2*

Malformation of cortical development (n=226)	*ACTB, ACTG1, ADGRG1, AHI1, AKT1, AKT3, AMPD2, AMT, AP4M1, ARFGEF2, ARL13B, ARX, ASNS, ASPM, ATP6V0A2, ATR, ATRX, B3GALNT2, B3GNT2, B4GAT1, B9D1, CASK, CC2D2A, CCND2, CDC6, CDK5, CDK5RAP2, CDKL5, CDON, CDT1, CENPJ, CEP135, CEP152, CEP290, CEP41, CEP63, CHMP1A, CLP1, CNTNAP2, COL18A1, COL4A1, CPLANE1, CREBBP, CUL4B, DCHS1, DCX, DEPDC5, DHCR24, DHCR7, DISP1, DLAT, DLD, DLL1, DYNC1H1, EFTUD2, EMX2, EOMES, ERMARD, ETFA, ETFB, ETFDH, EXOSC3, EZH2, FAT4, FGF8, FGFR3, FH, FKRP, FKTN, FLNA, FOXG1, FOXH1, GAS1, GCSH, GLDC, GLI2, GLI3, GMPPB, GNAQ, GPC3, GPSM2, HEPACAM, HESX1, HIP1, HSD17B4, IER3IP1, INPP5E, ISPD, KATNB1, KDM5C, WASHC5, KIF1BP, KIF11, KIF2A, KIF5C, KIF7, KNL1, L1CAM, LAMA2, LAMB1, LAMC3, LARGE1, MCPH1, MECP2, MED12, MEF2C, MIOS, MKS1, MRPS16, MTOR, MYCN, NBN, NDE1, NFIX, NHEJ1, NIPBL, NODAL, NPHP1, NPRL2, NPRL3, NRXN1, NSD1, NSDHL, OCLN, OFD1, OPHN1, ORC1, ORC4, ORC6, OTX2, PAFAH1B1, PAX6, PCDH19, PCNT, PDHA1, PDHB, PDHX, PDP1, PEX1, PEX10, PEX12, PEX13, PEX14, PEX16, PEX19, PEX2, PEX26, PEX3, PEX5, PHF6, PIEZO2, PIK3CA, PIK3R2, PNKP, POMGNT1, POMGNT2, POMK, POMT1, POMT2, PQBP1, PTCH1, PTEN, RAB18, RAB39B, RAB3GAP1, RAB3GAP2, RARS2, RBBP8, RELN, RIN2, RPGRIP1L, RTTN, SEC13, SEH1L, SEPSECS, SHH, SIX3, SLC12A6, SLC25A19, SLC35A2, SLC9A6, SNAP29, SOX2, SRD5A3, SRPX2, STIL, STRADA, TBC1D20, TCF4, TCTN1, TCTN2, TCTN3, TGIF1, TMEM138, TMEM216, TMEM231, TMEM237, RXYLT1, TMEM67, TSC1, TSC2, TSEN2, TSEN34, TSEN54, TTC21B, TUBA1A, TUBA8, TUBB, TUBB2A, TUBB2B, TUBB3, TUBB4A, TUBG1, TUBGCP6, UBE3A, UPF3B, VLDLR, VRK1, WDR24, WDR59, WDR62, YWHAE, YWHAG, ZEB2, ZIC2, ZNF423*

Clinical exome (n=4,872)	*A2M, A2ML1, A3GALT2, A4GALT, AAAS, AADAC, AAGAB, AARS, AARS2, AASS, AATF, ABAT, ABCA1, ABCA12, ABCA13, ABCA2, ABCA3, ABCA4, ABCA5, ABCA7, ABCA8, ABCB1, ABCB11, ABCB4, ABCB6, ABCB7, ABCC12, ABCC2, ABCC6, ABCC8, ABCC9, ABCD1, ABCD3, ABCD4, ABCG1, ABCG2, ABCG5, ABCG8, ABHD12, ABHD5, ABL1, ACACA, ACAD8, ACAD9, ACADL, ACADM, ACADS, ACADSB, ACADVL, ACAN, ACAT1, ACBD6, ACD, ACE, ACER3, ACHE, ACMSD, ACO2, ACOX1, ACOX2, ACP5, ACSF3, ACSL4, ACSS2, ACTA1, ACTA2, ACTB, ACTC1, ACTG1, ACTG2, ACTL6A, ACTL6B, ACTN1, ACTN2, ACTN4, ACTRT1, ACVR1, ACVR2B, ACVRL1, ACY1, ADA, ADAM10, ADAM17, ADAM22, ADAM9, ADAMTS1, ADAMTS10, ADAMTS13, ADAMTS17, ADAMTS18, ADAMTS2, ADAMTS3, ADAMTS9, ADAMTSL1, ADAMTSL2, ADAMTSL4, ADAR, ADAT3, ADCK3, ADCK4, ADCY1, ADCY10, ADCY3, ADCY5, ADCY6, ADD3, ADGRA3, ADGRB2, ADGRE2, ADGRG1, ADGRG2, ADGRG6, ADGRL2, ADGRV1, ADIPOQ, ADIPOR1, ADK, ADNP, ADORA1, ADPRHL2, ADRA2A, ADRA2B, ADRB2, ADSL, ADSSL1, AEBP1, AFF2, AFF4, AFG3L2, AFP, AGA, AGAP2, AGBL1, AGBL5, AGK, AGL, AGMO, AGPAT2, AGPS, AGRN, AGT, AGTR1, AGTR2, AGXT, AHCY, AHDC1, AHI1, AHR, AHSG, AHSP, AICDA, AIFM1, AIMP1, AIMP2, AIP, AIPL1, AIRE, AK1, AK2, AK7, AK9, AKAP2, AKAP9, AKR1B1, AKR1C2, AKR1C4, AKR1D1, AKR1E2, AKT1, AKT2, AKT3, ALAD, ALAS2, ALB, ALDH18A1, ALDH1A2, ALDH1A3, ALDH1B1, ALDH3A2, ALDH4A1, ALDH5A1, ALDH6A1, ALDH7A1, ALDOA, ALDOB, ALG1, ALG10B, ALG11, ALG12, ALG13, ALG14, ALG2, ALG3, ALG6, ALG8, ALG9, ALK, ALMS1, ALOX12B, ALOXE3, ALPI, ALPK3, ALPL, ALS2, ALX1, ALX3, ALX4, AMACR, AMBN, AMELX, AMER1, AMH, AMHR2, AMMECR1, AMN, AMPD1, AMPD2, AMPD3, AMT, AMZ2, ANG, ANGPT1, ANGPTL2, ANGPTL3, ANGPTL4, ANGPTL5, ANGPTL8, ANK1, ANK2, ANK3, ANKFY1, ANKH, ANKLE2, ANKRD1, ANKRD11, ANKRD26, ANKRD6, ANKS1A, ANKS3, ANKS6, ANLN, ANO10, ANO3, ANO5, ANO6, ANOS1, ANTXR1, ANTXR2, ANXA1, ANXA11, AP1S1, AP1S2, AP1S3, AP2S1, AP3B1, AP3B2, AP3D1, AP4B1, AP4E1, AP4M1, AP4S1, AP5Z1, APAF1, APBB1, APC, APC2, APCDD1, APEX1, APH1A, APOA1, APOA2, APOA4, APOA5, APOB, APOC2, APOC3, APOE, APOH, APOL1, APOPT1, APP, APPL1, APRT, APTX, AQP1, AQP2, AQP3, AQP5, AR, ARCN1, ARFGEF2, ARG1, ARHGAP24, ARHGAP29, ARHGAP31, ARHGAP32, ARHGAP4, ARHGAP5, ARHGDIA, ARHGEF10, ARHGEF15, ARHGEF18, ARHGEF2, ARHGEF28, ARHGEF6, ARHGEF9, ARID1A, ARID1B, ARID2, ARL13B, ARL14EP, ARL2BP, ARL3, ARL6, ARL6IP1, ARL6IP6, ARMC4, ARMC5, ARMC9, ARNT2, ARPC1B, ARR3, ARSA, ARSB, ARSE, ARSG, ARSI, ARV1, ARX, ASAH1, ASB10, ASCC1, ASCC3, ASCL1, ASGR1, ASH1L, ASL, ASMT, ASNS, ASPA, ASPH, ASPM, ASRGL1, ASS1, ASTN2, ASXL1, ASXL2, ASXL3, ATAD1, ATAD3A, ATCAY, ATF3, ATF4, ATF6, ATG12, ATG5, ATG7, ATIC, ATL1, ATL3, ATM, ATOH7, ATP10A, ATP11C, ATP13A1, ATP13A2, ATP13A3, ATP1A1, ATP1A2, ATP1A3, ATP2A1, ATP2A2, ATP2A3, ATP2B2, ATP2B3, ATP2B4, ATP2C1, ATP2C2, ATP4A, ATP5D, ATP5E, ATP6AP1, ATP6AP2, ATP6V0A2, ATP6V0A4, ATP6V0D2, ATP6V1A, ATP6V1B1, ATP6V1B2, ATP6V1E1, ATP6V1H, ATP7A, ATP7B, ATP8A2, ATP8B1, ATPAF2, ATR, ATRIP, ATRN, ATRX, ATXN2, AUH, AURKC, AUTS2, AVP, AVPR1A, AVPR2, AXIN1, AXIN2, AXL, B2M, B3GALNT1, B3GALNT2, B3GALT6, B3GAT3, B3GLCT, B4GALNT1, B4GALT1, B4GALT7, B4GAT1, B9D1, B9D2, BAAT, BACH2, BAG3, BANF1, BAP1, BARD1, BAZ1A, BAZ2B, BBIP1, BBS1, BBS10, BBS12, BBS2, BBS4, BBS5, BBS7, BBS9, BCAM, BCAP31, BCAT2, BCHE, BCKDHA, BCKDHB, BCKDK, BCL11A, BCL11B, BCL9, BCL9L, BCO1, BCOR, BCS1L, BEST1, BFSP1, BFSP2, BGN, BHLHA9, BHLHE41, BICC1, BICD2, BIN1, BLK, BLM, BLNK,*

BLOC1S3, BLOC1S6, BLVRA, BMP1, BMP10, BMP15, BMP2, BMP4, BMP6, BMP7, BMPER, BMPR1A, BMPR1B, BMPR2, BMS1, BNC1, BNC2, BOD1, BOLA3, BPGM, BPIFB6, BPTF, BRAF, BRAT1, BRCA1, BRCA2, BRD4, BRDT, BRF1, BRIP1, BRPF1, BRWD3, BSCL2, BSN, BSND, BTAF1, BTD, BTG2, BTK, BUB1, BUB1B, BUB3, BVES, C11orf70, C11orf80, C12orf4, C12orf57, C12orf65, C14orf93, C15orf41, C19orf12, C19orf70, C1QA, C1QB, C1QC, C1QTNF5, C1R, C1S, C2, C21orf2, C21orf59, C2CD3, C2orf71, C3, C3AR1, C3orf67, C4A, C4B, C5, C5AR2, C5orf42, C6, C7, C8A, C8B, C8orf37, C9, C9orf72, CA1, CA12, CA2, CA4, CA5A, CA8, CABIN1, CABP2, CABP4, CACNA1A, CACNA1C, CACNA1D, CACNA1E, CACNA1F, CACNA1G, CACNA1H, CACNA1S, CACNA2D1, CACNA2D2, CACNA2D3, CACNA2D4, CACNB1, CACNB2, CACNB4, CACNG2, CAD, CADM1, CADPS2, CALCB, CALCRL, CALHM1, CALM1, CALM2, CALM3, CALR, CALR3, CAMK2A, CAMK2B, CAMK4, CAMTA1, CAMTA2, CANT1, CAPN1, CAPN10, CAPN12, CAPN3, CAPN5, CAPRIN1, CARD10, CARD11, CARD14, CARD9, CARMIL2, CARS2, CARTPT, CASK, CASP10, CASP14, CASP2, CASP3, CASP8, CASP9, CASQ1, CASQ2, CASR, CAST, CASZ1, CAT, CATSPER1, CAV1, CAV3, CBL, CBLB, CBS, CC2D1A, CC2D2A, CCAR2, CCBE1, CCDC103, CCDC114, CCDC115, CCDC141, CCDC151, CCDC174, CCDC22, CCDC39, CCDC40, CCDC47, CCDC50, CCDC65, CCDC78, CCDC8, CCDC82, CCDC88A, CCDC88C, CCER2, CCM2, CCNA2, CCND2, CCNF, CCNK, CCNO, CCS, CCT2, CCT4, CCT5, CCT7, CD151, CD163L1, CD164, CD177, CD19, CD207, CD247, CD27, CD2AP, CD300LF, CD320, CD36, CD3D, CD3E, CD3G, CD40, CD40LG, CD46, CD55, CD59, CD70, CD79A, CD79B, CD81, CD8A, CD96, CDAN1, CDC14A, CDC42, CDC42BPB, CDC45, CDC5L, CDC6, CDC73, CDCA7, CDCA8, CDH1, CDH11, CDH15, CDH16, CDH2, CDH23, CDH3, CDHR1, CDK10, CDK13, CDK4, CDK5, CDK5R1, CDK5RAP2, CDK6, CDK9, CDKL5, CDKN1A, CDKN1B, CDKN1C, CDKN2A, CDKN2B, CDKN2C, CDON, CDSN, CDT1, CDX1, CDX2, CEACAM16, CEBPA, CEBPE, CECR1, CEL, CELF4, CELSR1, CELSR2, CELSR3, CEMIP, CENPE, CENPF, CENPJ, CENPT, CEP104, CEP120, CEP135, CEP152, CEP164, CEP19, CEP250, CEP290, CEP41, CEP55, CEP57, CEP63, CEP78, CEP83, CEP97, CERKL, CERS1, CERS3, CES1, CETP, CFAP43, CFAP44, CFAP53, CFAP69, CFB, CFC1, CFD, CFH, CFHR1, CFHR2, CFHR3, CFHR5, CFI, CFL2, CFP, CFTR, CGNL1, CHAF1B, CHAMP1, CHAT, CHCHD10, CHCHD2, CHD1, CHD1L, CHD2, CHD3, CHD4, CHD7, CHD8, CHEK2, CHIT1, CHKB, CHM, CHMP1A, CHMP2B, CHMP4B, CHN1, CHRDL1, CHRM2, CHRM3, CHRNA1, CHRNA2, CHRNA4, CHRNA7, CHRNB1, CHRNB2, CHRNB3, CHRND, CHRNE, CHRNG, CHST11, CHST14, CHST3, CHST6, CHSY1, CHUK, CIB1, CIB2, CIB3, CIC, CIDEC, CIITA, CISD2, CIT, CITED2, CIZ1, CKAP2L, CKM, CLASP1, CLCA2, CLCF1, CLCN1, CLCN2, CLCN4, CLCN5, CLCN7, CLCNKA, CLCNKB, CLDN1, CLDN10, CLDN14, CLDN16, CLDN19, CLEC16A, CLEC4C, CLHC1, CLIC5, CLIP1, CLMN, CLMP, CLN3, CLN5, CLN6, CLN8, CLP1, CLPB, CLPP, CLPX, CLRN1, CLTC, CLTCL1, CLU, CLUAP1, CNGA1, CNGA2, CNGA3, CNGB1, CNGB3, CNKSR1, CNKSR2, CNNM2, CNNM4, CNOT3, CNPY3, CNR1, CNTN1, CNTN2, CNTN3, CNTN4, CNTN5, CNTN6, CNTNAP1, CNTNAP2, CNTNAP3, CNTNAP4, CNTNAP5, CNTRL, CNTROB, COA3, COA5, COA6, COA7, COASY, COCH, COG1, COG2, COG4, COG5, COG6, COG7, COG8, COL10A1, COL11A1, COL11A2, COL12A1, COL13A1, COL14A1, COL17A1, COL18A1, COL1A1, COL1A2, COL25A1, COL27A1, COL2A1, COL3A1, COL4A1, COL4A2, COL4A3, COL4A4, COL4A5, COL4A6, COL5A1, COL5A2, COL6A1, COL6A2, COL6A3, COL6A5, COL6A6, COL7A1, COL8A2, COL9A1, COL9A2, COL9A3, COLEC10, COLEC11, COLGALT1,

COLQ, COMP, COMT, COPA, COPB2, COQ2, COQ4, COQ5, COQ6, COQ7, COQ9, CORIN, CORO1A, COX10, COX14, COX15, COX20, COX4I1, COX4I2, COX5A, COX6A1, COX6B1, COX7B, COX8A, CP, CPA1, CPA6, CPAMD8, CPB1, CPLX1, CPN1, CPOX, CPS1, CPT1A, CPT1C, CPT2, CR1, CR2, CRADD, CRAT, CRB1, CRB2, CRBN, CREB1, CREB3L1, CREB3L3, CREBBP, CRELD1, CRH, CRIPT, CRLF1, CRTAP, CRX, CRY1, CRY2, CRYAA, CRYAB, CRYBA1, CRYBA2, CRYBA4, CRYBB1, CRYBB2, CRYBB3, CRYGA, CRYGB, CRYGC, CRYGD, CRYGS, CRYM, CSF1R, CSF2RA, CSF2RB, CSF3R, CSGALNACT1, CSMD1, CSNK1D, CSNK2A1, CSNK2B, CSPP1, CSRP1, CSRP3, CST3, CSTA, CSTB, CTBP1, CTC1, CTCF, CTDP1, CTF1, CTH, CTHRC1, CTLA4, CTNNA1, CTNNA2, CTNNA3, CTNNB1, CTNND1, CTNND2, CTNS, CTPS1, CTR9, CTRC, CTSA, CTSB, CTSC, CTSD, CTSF, CTSH, CTSK, CTTNBP2, CTU2, CUBN, CUL3, CUL4B, CUL7, CUX1, CWC27, CWF19L1, CXCR1, CXCR2, CXCR4, CXorf56, CYB561, CYB5A, CYB5R3, CYBA, CYBB, CYBRD1, CYC1, CYCS, CYFIP1, CYLD, CYP11A1, CYP11B1, CYP11B2, CYP17A1, CYP19A1, CYP1B1, CYP21A2, CYP24A1, CYP26B1, CYP26C1, CYP27A1, CYP27B1, CYP2C8, CYP2D6, CYP2F1, CYP2R1, CYP2U1, CYP3A43, CYP3A7, CYP4F22, CYP4V2, CYP51A1, CYP7A1, CYP7B1, D2HGDH, DACH1, DACT1, DAG1, DAO, DAPK1, DAPK3, DAPP1, DARS, DARS2, DAW1, DAZL, DBH, DBR1, DBT, DCAF17, DCAF8, DCC, DCDC2, DCHS1, DCLRE1C, DCN, DCPS, DCTN1, DCX, DCXR, DDB2, DDC, DDHD1, DDHD2, DDOST, DDR2, DDRGK1, DDX11, DDX24, DDX3X, DDX41, DDX58, DDX59, DEAF1, DECR1, DEFA4, DENND4B, DENND5A, DENR, DEPDC5, DES, DFNB59, DGAT1, DGAT2, DGCR2, DGKE, DGUOK, DHCR24, DHCR7, DHDDS, DHFR, DHH, DHODH, DHTKD1, DHX30, DHX32, DHX38, DIABLO, DIAPH1, DIAPH2, DIAPH3, DICER1, DIP2A, DIP2B, DIP2C, DIS3L2, DISC1, DISP1, DKC1, DLAT, DLC1, DLD, DLG3, DLG4, DLGAP1, DLGAP2, DLGAP3, DLL1, DLL3, DLL4, DLX3, DLX4, DLX5, DLX6, DMBX1, DMC1, DMD, DMGDH, DMP1, DMPK, DMRT1, DMRTA2, DMXL2, DNA2, DNAAF1, DNAAF2, DNAAF3, DNAAF5, DNAH1, DNAH10, DNAH11, DNAH5, DNAH6, DNAH9, DNAI1, DNAI2, DNAJB11, DNAJB13, DNAJB2, DNAJB6, DNAJC12, DNAJC13, DNAJC17, DNAJC19, DNAJC21, DNAJC3, DNAJC5, DNAJC6, DNAL1, DNAL4, DNASE1, DNASE1L3, DNASE2, DND1, DNM1, DNM1L, DNM2, DNMBP, DNMT1, DNMT3A, DNMT3B, DOCK2, DOCK3, DOCK6, DOCK7, DOCK8, DOK7, DOLK, DONSON, DPAGT1, DPF2, DPH1, DPM1, DPM2, DPM3, DPP10, DPP6, DPT, DPY19L2, DPYD, DPYS, DPYSL2, DRAM2, DRC1, DRD2, DROSHA, DRP2, DSC2, DSC3, DSCAM, DSE, DSG1, DSG2, DSG4, DSP, DSPP, DST, DSTYK, DTNA, DTNBP1, DUOX1, DUOX2, DUOXA2, DUT, DVL1, DVL3, DYM, DYNC1H1, DYNC2H1, DYNC2LI1, DYRK1A, DYRK1B, DYSF, DYX1C1, DZIP1L, EARS2, EBF2, EBF3, EBP, ECE1, ECEL1, ECHS1, ECM1, EDA, EDA2R, EDAR, EDARADD, EDC3, EDN1, EDN3, EDNRA, EDNRB, EED, EEF1A2, EEF1B2, EEF1D, EEF2, EFEMP1, EFEMP2, EFHC1, EFL1, EFNA4, EFNB1, EFNB2, EFR3A, EFTUD2, EGF, EGFR, EGLN1, EGLN2, EGR2, EHHADH, EHMT1, EIF2AK3, EIF2AK4, EIF2B1, EIF2B2, EIF2B3, EIF2B4, EIF2B5, EIF2S3, EIF3F, EIF4A2, EIF4A3, EIF4E, EIF4ENIF1, EIF4G1, ELAC2, ELANE, ELAVL3, ELF2, ELK1, ELMO2, ELMOD3, ELN, ELOVL1, ELOVL4, ELOVL5, ELP2, ELP4, EMC1, EMD, EMG1, EMILIN1, EML1, EMP2, EMSY, EMX2, ENAH, ENAM, ENG, ENHO, ENO2, ENO3, ENPP1, ENTPD1, EOGT, EOMES, EP300, EP400, EPAS1, EPB41, EPB41L1, EPB41L4A, EPB42, EPCAM, EPG5, EPHA2, EPHA4, EPHB2, EPHB4, EPHX1, EPM2A, EPO, EPOR, EPRS, EPS15L1, EPS8, EPS8L2, EPS8L3, EPX, ERAL1, ERBB2, ERBB3, ERBB4, ERBIN, ERCC1, ERCC2, ERCC3, ERCC4, ERCC5, ERCC6, ERCC6L2, ERCC8, ERF, ERGIC1, ERLIN1, ERLIN2, ERMARD,

ESCO2, ESPN, ESR1, ESR2, ESRP1, ESRP2, ESRRA, ESRRB, ETFA, ETFB, ETFDH, ETHE1, ETV4, ETV6, EVC, EVC2, EWSR1, EXO1, EXOC3L2, EXOC6B, EXOC8, EXOSC2, EXOSC3, EXOSC8, EXOSC9, EXPH5, EXT1, EXT2, EXTL3, EYA1, EYA4, EYS, EZH1, EZH2, EZR, F10, F11, F12, F13A1, F13B, F2, F2R, F5, F7, F8, F9, FA2H, FAAH, FAAH2, FAAP24, FABP3, FABP7, FADD, FAF1, FAH, FAM111A, FAM111B, FAM120AOS, FAM126A, FAM136A, FAM151A, FAM160A1, FAM161A, FAM177A1, FAM20A, FAM20C, FAM234B, FAM46A, FAM83G, FAM83H, FAM92B, FAN1, FANCA, FANCB, FANCC, FANCD2, FANCE, FANCF, FANCG, FANCI, FANCL, FANCM, FAR1, FARS2, FARSB, FAS, FASLG, FASN, FASTKD2, FAT1, FAT2, FAT3, FAT4, FBLIM1, FBLN1, FBLN2, FBLN5, FBN1, FBN2, FBN3, FBP1, FBXL4, FBXO11, FBXO25, FBXO28, FBXO31, FBXO32, FBXO38, FBXO7, FBXW4, FCGR1A, FCGR3A, FCN1, FCN2, FDFT1, FDPS, FDX1L, FDXR, FECH, FEN1, FERMT1, FERMT3, FEZF1, FGA, FGB, FGD1, FGD4, FGF10, FGF12, FGF14, FGF16, FGF17, FGF20, FGF23, FGF3, FGF5, FGF8, FGF9, FGFBP1, FGFR1, FGFR2, FGFR3, FGG, FH, FHL1, FHL2, FHOD3, FIBP, FIG4, FIGLA, FITM2, FKBP10, FKBP14, FKBP5, FKBP6, FKBPL, FKRP, FKTN, FLAD1, FLCN, FLG, FLG2, FLI1, FLNA, FLNB, FLNC, FLRT1, FLT1, FLT3, FLT4, FLVCR1, FLVCR2, FMN2, FMO3, FMR1, FN1, FN3K, FNIP1, FOLR1, FOXA2, FOXA3, FOXC1, FOXC2, FOXD4, FOXE1, FOXE3, FOXF1, FOXG1, FOXH1, FOXI1, FOXL2, FOXN1, FOXP1, FOXP2, FOXP3, FOXRED1, FRAS1, FREM1, FREM2, FRMD4A, FRMD7, FRMPD4, FRRS1L, FRY, FRZB, FSCN2, FSHB, FSHR, FSIP2, FTCD, FTH1, FTL, FTMT, FTO, FTSJ1, FUCA1, FUS, FUT1, FUT2, FUT7, FUT8, FUZ, FXN, FXYD2, FYCO1, FZD2, FZD4, FZD5, FZD6, G6PC, G6PC3, G6PD, GAA, GAB1, GABBR2, GABRA1, GABRA2, GABRA3, GABRA5, GABRA6, GABRB1, GABRB2, GABRB3, GABRD, GABRE, GABRG2, GAD1, GAL, GAL3ST2, GAL3ST4, GALC, GALE, GALK1, GALNS, GALNT12, GALNT14, GALNT2, GALNT3, GALNTL5, GALR2, GALT, GAMT, GAN, GANAB, GAP43, GAPVD1, GARS, GAS1, GAS8, GATA1, GATA2, GATA3, GATA4, GATA5, GATA6, GATAD1, GATAD2B, GATB, GATC, GATM, GBA, GBA2, GBE1, GCDH, GCGR, GCH1, GCK, GCKR, GCLC, GCM2, GCNT2, GCSH, GDAP1, GDAP2, GDF1, GDF2, GDF3, GDF5, GDF6, GDF9, GDI1, GDNF, GEMIN4, GEN1, GFAP, GFER, GFI1, GFI1B, GFM1, GFM2, GFPT1, GGCX, GGNBP2, GH1, GH2, GHR, GHRHR, GHSR, GIF, GIGYF1, GIGYF2, GINS1, GIPC3, GIT1, GJA1, GJA3, GJA5, GJA8, GJB1, GJB2, GJB3, GJB4, GJB6, GJC1, GJC2, GJC3, GK, GLA, GLB1, GLDC, GLDN, GLE1, GLI1, GLI2, GLI3, GLIS2, GLIS3, GLMN, GLP1R, GLRA1, GLRA2, GLRB, GLRX5, GLS, GLUD1, GLUL, GLYCTK, GM2A, GMNN, GMPPA, GMPPB, GNA11, GNAI3, GNAL, GNAO1, GNAQ, GNAS, GNAT1, GNAT2, GNB1, GNB1L, GNB2, GNB3, GNB4, GNB5, GNE, GNMT, GNPAT, GNPTAB, GNPTG, GNRH1, GNRHR, GNS, GOLGA2, GON4L, GORAB, GOSR2, GOT1, GP1BA, GP1BB, GP6, GP9, GPAA1, GPATCH3, GPATCH8, GPBAR1, GPC3, GPC4, GPC6, GPD1, GPD1L, GPD2, GPHN, GPI, GPIHBP1, GPKOW, GPNMB, GPR143, GPR161, GPR179, GPR68, GPR85, GPR88, GPRASP2, GPSM2, GPT, GPT2, GPX4, GRAMD1B, GREB1L, GREM1, GREM2, GRHL2, GRHL3, GRHPR, GRIA1, GRIA3, GRIA4, GRID1, GRID2, GRIK2, GRIK5, GRIN1, GRIN2A, GRIN2B, GRIN2D, GRIP1, GRK1, GRK5, GRM1, GRM4, GRM6, GRM7, GRN, GRXCR1, GRXCR2, GSC, GSN, GSPT2, GSR, GSS, GTF2E2, GTF2H5, GTPBP2, GTPBP3, GUCA1A, GUCA1B, GUCY1A3, GUCY2C, GUCY2D, GUF1, GUSB, GYG1, GYG2, GYPA, GYPB, GYPC, GYS1, GYS2, GZF1, H19, H6PD, HAAO, HACD1, HACE1, HADH, HADHA, HADHB, HAL, HAMP, HAND1, HAND2, HAO1, HARS, HARS2, HAS2, HAUS7, HAVCR2, HAX1, HBA1, HBA2, HBB, HBD, HBG1, HBG2, HBM, HBS1L, HCCS, HCFC1, HCN1, HCN2, HCN4,

HCRT, HCRTR2, HDAC4, HDAC6, HDAC8, HDC, HECTD4, HECW2, HELLS, HELZ, HEPACAM, HEPHL1, HERC1, HERC2, HES7, HESX1, HEXA, HEXB, HFE, HFE2, HFM1, HGD, HGF, HGSNAT, HHAT, HHIP, HIBCH, HIKESHI, HINT1, HIP1, HIST1H1E, HIST1H4B, HIST1H4C, HIST3H3, HIVEP2, HIVEP3, HK1, HK2, HLA-A, HLA-C, HLCS, HLX, HMBS, HMCN1, HMG20A, HMGA1, HMGA2, HMGB3, HMGCL, HMGCS2, HMGN1, HMOX1, HMX1, HNF1A, HNF1B, HNF4A, HNMT, HNRNPA0, HNRNPA1, HNRNPA2B1, HNRNPDL, HNRNPH1, HNRNPH2, HNRNPK, HNRNPU, HOGA1, HOMER2, HOMEZ, HOOK1, HOXA1, HOXA10, HOXA11, HOXA13, HOXA2, HOXA4, HOXB1, HOXB13, HOXB6, HOXC13, HOXD10, HOXD11, HOXD13, HOXD4, HP, HPCA, HPD, HPGD, HPRT1, HPS1, HPS3, HPS4, HPS5, HPS6, HPSE2, HR, HRAS, HRG, HS6ST1, HSD11B1, HSD11B2, HSD17B10, HSD17B3, HSD17B4, HSD3B2, HSD3B7, HSF2, HSF4, HSPA1L, HSPA9, HSPB1, HSPB3, HSPB6, HSPB8, HSPD1, HSPG2, HTR1A, HTR2A, HTR2B, HTRA1, HTRA2, HUWE1, HYAL1, HYAL2, HYDIN, HYLS1, HYOU1, HYPK, IAPP, IARS, IARS2, IBA57, ICE2, ICK, ICOS, ICOSLG, IDH1, IDH2, IDH3A, IDS, IDUA, IER3IP1, IFIH1, IFITM5, IFNAR2, IFNGR1, IFNGR2, IFNLR1, IFT122, IFT140, IFT172, IFT27, IFT43, IFT52, IFT57, IFT80, IFT81, IFT88, IGBP1, IGF1, IGF1R, IGF2, IGFALS, IGFBP6, IGFBP7, IGHMBP2, IGLL1, IGSF1, IGSF10, IGSF3, IHH, IKBKAP, IKBKB, IKBKG, IKZF1, IL10, IL10RA, IL10RB, IL11, IL11RA, IL12B, IL12RB1, IL12RB2, IL17F, IL17RA, IL17RC, IL17RD, IL1RAPL1, IL1RN, IL21, IL21R, IL23R, IL27RA, IL2RA, IL2RG, IL31RA, IL36RN, IL6, IL6ST, IL7R, ILDR1, ILF2, ILK, IMPA1, IMPAD1, IMPDH1, IMPG1, IMPG2, INF2, INHA, INHBA, INIP, INO80, INO80D, INPP4A, INPP5B, INPP5E, INPP5K, INPPL1, INS, INSL3, INSR, INTS1, INTS6, INTS8, INTU, INVS, IPMK, IQCB1, IQCK, IQSEC1, IQSEC2, IRAK3, IRAK4, IRF2BP2, IRF2BPL, IRF3, IRF4, IRF6, IRF7, IRF8, IRF9, IRS1, IRS4, IRX5, ISCA1, ISCA2, ISCU, ISG15, ISL1, ISLR2, ISPD, ITCH, ITGA2, ITGA2B, ITGA3, ITGA4, ITGA6, ITGA7, ITGA8, ITGA9, ITGB2, ITGB3, ITGB4, ITGB6, ITK, ITM2B, ITPA, ITPR1, ITPR2, ITSN1, ITSN2, IVD, IYD, JAG1, JAGN1, JAK1, JAK2, JAK3, JAKMIP1, JAM3, JARID2, JPH1, JPH2, JUP, KANK1, KANK2, KANK4, KANSL1, KARS, KAT2B, KAT6A, KAT6B, KATNAL2, KATNB1, KBTBD13, KCNA1, KCNA2, KCNA4, KCNA5, KCNAB1, KCNAB2, KCNB1, KCNB2, KCNC1, KCNC3, KCND2, KCND3, KCNE1, KCNE2, KCNE3, KCNE4, KCNE5, KCNG1, KCNH1, KCNH2, KCNH7, KCNJ1, KCNJ10, KCNJ11, KCNJ13, KCNJ16, KCNJ18, KCNJ2, KCNJ5, KCNJ6, KCNJ8, KCNK17, KCNK18, KCNK3, KCNK4, KCNK9, KCNMA1, KCNN3, KCNN4, KCNQ1, KCNQ2, KCNQ3, KCNQ4, KCNQ5, KCNS2, KCNT1, KCNT2, KCNV2, KCTD1, KCTD17, KCTD3, KCTD7, KDELC2, KDF1, KDM1A, KDM3A, KDM5A, KDM5B, KDM5C, KDM6A, KDM6B, KDR, KDSR, KEAP1, KERA, KHDC3L, KHK, KIAA0556, KIAA0586, KIAA0753, KIAA1109, KIAA1279, KIAA1549, KIAA1586, KIAA1715, KIDINS220, KIF11, KIF14, KIF15, KIF17, KIF1A, KIF1B, KIF1C, KIF20A, KIF21A, KIF22, KIF23, KIF26B, KIF2A, KIF4A, KIF5A, KIF5C, KIF7, KIRREL3, KISS1, KISS1R, KIT, KITLG, KIZ, KL, KLB, KLC4, KLF1, KLF10, KLF11, KLF8, KLHL10, KLHL15, KLHL24, KLHL3, KLHL40, KLHL41, KLHL7, KLHL9, KLK12, KLK4, KLKB1, KMT2A, KMT2B, KMT2C, KMT2D, KMT2E, KMT5B, KNG1, KNL1, KPNA7, KPTN, KRAS, KREMEN1, KRIT1, KRT1, KRT10, KRT12, KRT13, KRT14, KRT16, KRT17, KRT18, KRT2, KRT25, KRT3, KRT4, KRT5, KRT6A, KRT6B, KRT6C, KRT71, KRT74, KRT8, KRT81, KRT83, KRT85, KRT86, KRT9, KSR2, KY, KYNU, L1CAM, L2HGDH, LACC1, LAGE3, LAMA1, LAMA2, LAMA3, LAMA4, LAMA5, LAMB1, LAMB2, LAMB3, LAMC1, LAMC2, LAMC3, LAMP2, LAMTOR2, LARGE1, LARP7, LARS, LARS2, LAS1L, LAT, LBR, LBX2, LCA5, LCAT, LCK, LCT, LDB3, LDHA, LDHB,

LDLR, LDLRAP1, LEFTY2, LEMD2, LEMD3, LENG8, LEO1, LEP, LEPR, LFNG, LGI1, LGI4, LHB, LHCGR, LHFPL5, LHX1, LHX3, LHX4, LIAS, LIF, LIFR, LIG1, LIG4, LIM2, LIMA1, LIMS2, LIN7B, LINGO1, LINS1, LIPA, LIPC, LIPE, LIPG, LIPH, LIPI, LIPN, LIPT1, LIPT2, LITAF, LMAN1, LMAN2L, LMBR1, LMBRD1, LMF1, LMNA, LMNB2, LMOD1, LMOD3, LMX1A, LMX1B, LONP1, LOR, LOX, LOXHD1, LOXL2, LOXL3, LPA, LPAR6, LPIN1, LPIN2, LPIN3, LPL, LRAT, LRBA, LRIG2, LRIT3, LRP1, LRP10, LRP2, LRP4, LRP5, LRP6, LRPAP1, LRPPRC, LRRC10, LRRC56, LRRC6, LRRIQ3, LRRK1, LRRK2, LRRTM4, LRSAM1, LRTOMT, LSS, LTBP2, LTBP3, LTBP4, LY96, LYN, LYRM4, LYRM7, LYST, LYZ, LZTFL1, LZTR1, MAB21L2, MACF1, MACROD2, MAD2L2, MAF, MAFA, MAFB, MAG, MAGED2, MAGEL2, MAGI2, MAGT1, MAK, MALT1, MAMLD1, MAN1B1, MAN2B1, MANBA, MAOA, MAOB, MAP1B, MAP2K1, MAP2K2, MAP2K5, MAP3K1, MAP3K14, MAP3K6, MAP3K7, MAP4, MAP4K4, MAPK10, MAPK8IP1, MAPKAP1, MAPKAPK3, MAPKBP1, MAPRE2, MAPT, MARK4, MARS, MARS2, MARVELD2, MASP1, MAST1, MASTL, MAT1A, MAT2A, MATN3, MATN4, MATR3, MAX, MBD1, MBD4, MBD5, MBNL3, MBOAT7, MBTPS2, MC1R, MC2R, MC3R, MC4R, MCCC1, MCCC2, MCEE, MCF2L, MCFD2, MCHR1, MCIDAS, MCM2, MCM3AP, MCM4, MCM5, MCM8, MCM9, MCOLN1, MCPH1, MCTP2, MDH2, MECOM, MECP2, MECR, MED12, MED13, MED13L, MED17, MED20, MED23, MED25, MEF2A, MEF2C, MEFV, MEGF10, MEGF8, MEI1, MEIOB, MEIS2, MEN1, MEOX1, MEOX2, MERTK, MESP1, MESP2, MET, METTL23, METTL5, MFAP5, MFF, MFGE8, MFN2, MFRP, MFSD2A, MFSD8, MGAT2, MGLL, MGME1, MGMT, MGP, MGST2, MIA, MIB1, MICA, MICAL1, MICU1, MICU2, MID1, MID2, MIEF2, MINPP1, MIP, MIPEP, MIR125A, MIR137, MIR16-1, MIR17, MIR17HG, MIR184, MIR204, MIR2861, MIR29B2, MIR30C1, MIR372, MIR96, MITF, MKKS, MKL1, MKRN3, MKS1, MLC1, MLH1, MLH3, MLPH, MLYCD, MMAA, MMAB, MMACHC, MMADHC, MME, MMP13, MMP14, MMP2, MMP20, MMP21, MMP9, MNS1, MNX1, MOCOS, MOCS1, MOCS2, MOCS3, MOG, MOGS, MORC2, MPC1, MPDU1, MPDZ, MPEG1, MPI, MPL, MPLKIP, MPO, MPV17, MPZ, MPZL2, MRAP, MRAP2, MRAS, MRE11A, MRI1, MRM2, MRPL10, MRPL12, MRPL3, MRPL43, MRPL44, MRPS16, MRPS2, MRPS22, MRPS23, MRPS34, MRPS7, MS4A1, MS4A2, MSH2, MSH3, MSH4, MSH5, MSH6, MSL3, MSMO1, MSN, MSR1, MSRB3, MST1R, MSTN, MSTO1, MSX1, MSX2, MTAP, MTF1, MTFMT, MTHFD1, MTHFR, MTHFS, MTM1, MTMR14, MTMR2, MTNR1A, MTNR1B, MTO1, MTOR, MTPAP, MTR, MTRR, MTSS1L, MTTP, MURC, MUSK, MUT, MUTYH, MVD, MVK, MXRA8, MYBPC1, MYBPC3, MYBPHL, MYCBP2, MYCN, MYD88, MYF5, MYF6, MYH1, MYH10, MYH11, MYH14, MYH2, MYH3, MYH6, MYH7, MYH8, MYH9, MYL1, MYL2, MYL3, MYL4, MYLIP, MYLK, MYLK2, MYLK3, MYO15A, MYO18B, MYO1A, MYO1E, MYO1H, MYO3A, MYO5A, MYO5B, MYO6, MYO7A, MYO9A, MYO9B, MYOC, MYOCD, MYOD1, MYOM1, MYOT, MYOZ2, MYPN, MYRF, MYT1, MYT1L, NAA10, NAA15, NAA20, NAA25, NAA30, NAA35, NAA38, NAA40, NAA50, NAA60, NACC1, NADK2, NAF1, NAGA, NAGLU, NAGPA, NAGS, NALCN, NANOS1, NANOS3, NANS, NAPB, NARFL, NARS2, NAT8L, NAV2, NAXD, NAXE, NBAS, NBEA, NBEAL2, NBN, NCAPD2, NCAPD3, NCAPG2, NCAPH, NCF1, NCF2, NCF4, NCKAP1, NCOA6, NCOR1, NCS1, NCSTN, NDE1, NDP, NDRG1, NDRG4, NDST1, NDUFA1, NDUFA10, NDUFA11, NDUFA12, NDUFA13, NDUFA2, NDUFA4, NDUFA8, NDUFA9, NDUFAF1, NDUFAF2, NDUFAF3, NDUFAF4, NDUFAF5, NDUFAF6, NDUFB10, NDUFB11, NDUFB3, NDUFB4, NDUFB8, NDUFB9, NDUFS1, NDUFS2, NDUFS3, NDUFS4, NDUFS6, NDUFS7, NDUFS8, NDUFV1, NDUFV2, NEB, NEBL, NECAP1, NECTIN1, NECTIN4, NEDD4L, NEFH, NEFL, NEFM, NEIL1, NEK1,

NEK11, NEK2, NEK8, NEK9, NEU1, NEURL4, NEUROD1, NEUROG3, NEXN, NF1, NF2, NFASC, NFATC1, NFE2L2, NFIA, NFIB, NFIL3, NFIX, NFKB1, NFKB2, NFKBIA, NFS1, NFU1, NGF, NGLY1, NHEJ1, NHLRC1, NHLRC2, NHP2, NHS, NID1, NIN, NINL, NIPA1, NIPA2, NIPAL4, NIPBL, NKX2-1, NKX2-2, NKX2-5, NKX2-6, NKX3-2, NKX6-1, NKX6-2, NLGN1, NLGN2, NLGN3, NLGN4X, NLGN4Y, NLRC4, NLRP1, NLRP12, NLRP2, NLRP3, NLRP5, NLRP7, NME7, NME8, NMNAT1, NNT, NOBOX, NOD2, NODAL, NOG, NOL3, NONO, NOP10, NOP14, NOS1, NOS1AP, NOS3, NOTCH1, NOTCH2, NOTCH3, NOTCH4, NPAS2, NPAT, NPC1, NPC1L1, NPC2, NPEPPS, NPHP1, NPHP3, NPHP4, NPHS1, NPHS2, NPL, NPPA, NPPC, NPR2, NPR3, NPRL2, NPRL3, NPY4R, NQO2, NR0B1, NR0B2, NR1D2, NR1H3, NR1H4, NR1I2, NR1I3, NR2E3, NR2F1, NR2F2, NR3C1, NR3C2, NR4A2, NR5A1, NRAP, NRAS, NRDC, NRG1, NRG4, NRGN, NRIP1, NRL, NRP1, NRTN, NRXN1, NRXN2, NRXN3, NSD1, NSDHL, NSMCE2, NSMCE3, NSMF, NSUN2, NSUN3, NSUN7, NT5C2, NT5C3A, NT5E, NTF4, NTHL1, NTRK1, NTRK2, NTRK3, NUAK1, NUB1, NUBPL, NUDC, NUP107, NUP133, NUP155, NUP188, NUP205, NUP37, NUP43, NUP62, NUP88, NUP93, NUS1, NXF5, NXNL1, NXPH3, NYX, OAS1, OAT, OBFC1, OBSCN, OBSL1, OCA2, OCLN, OCRL, ODC1, OFD1, OGG1, OGT, OLFM2, OMG , OOEP, OPA1, OPA3, OPHN1, OPLAH, OPN1LW, OPN1MW, OPN1SW, OPTC, OPTN, OR52M1, OR6C75, ORC1, ORC4, ORC6, OSBPL2, OSGEP, OSMR, OSTM1, OTC, OTOA, OTOF, OTOG, OTOGL, OTOR, OTUD1, OTUD4, OTUD6B, OTUD7A, OTULIN, OTX2, OVOL2, OXA1L, OXCT1, OXTR, P2RX1, P2RX2, P2RX5, P2RY11, P2RY12, P3H1, P3H2, P4HA1, P4HA2, P4HB, PABPN1, PACS1, PACS2, PADI3, PADI6, PAFAH1B1, PAFAH1B2, PAH, PAK1, PAK3, PALB2, PAM16, PAN2, PANK2, PANX1, PAPPA2, PAPSS2, PARD3B, PARK2, PARK7, PARL, PARN, PARP1, PARP10, PARS2, PASK, PATL2, PAX1, PAX2, PAX3, PAX4, PAX5, PAX6, PAX7, PAX8, PAX9, PAXBP1, PBX1, PC, PCBD1, PCCA, PCCB, PCDH12, PCDH15, PCDH19, PCDHB4, PCDHGA4, PCGF2, PCK1, PCLO, PCM1, PCNA, PCNT, PCSK1, PCSK7, PCSK9, PCYT1A, PDCD10, PDE10A, PDE11A, PDE1C, PDE2A, PDE3A, PDE4D, PDE5A, PDE6A, PDE6B, PDE6C, PDE6D, PDE6G, PDE6H, PDE8B, PDGFB, PDGFRA, PDGFRB, PDHA1, PDHA2, PDHB, PDHX, PDK3, PDLIM3, PDP1, PDSS1, PDSS2, PDX1, PDYN, PDZD7, PECR, PEPD, PER1, PER2, PER3, PET100, PET117, PEX1, PEX10, PEX12, PEX13, PEX14, PEX16, PEX19, PEX2, PEX26, PEX3, PEX5, PEX6, PEX7, PFKM, PFN1, PGAM1, PGAM2, PGAP1, PGAP2, PGAP3, PGK1, PGM1, PGM3, PGR, PGRMC1, PHACTR1, PHB, PHC1, PHEX, PHF2, PHF21A, PHF3, PHF6, PHF8, PHGDH, PHIP, PHKA1, PHKA2, PHKB, PHKG2, PHLDB3, PHOX2A, PHOX2B, PHRF1, PHYH, PHYKPL, PI4K2A, PI4KA, PIBF1, PICK1, PIEZO1, PIEZO2, PIF1, PIGA, PIGC, PIGG, PIGH, PIGL, PIGM, PIGN, PIGO, PIGP, PIGQ, PIGS, PIGT, PIGV, PIGW, PIGY, PIH1D3, PIK3CA, PIK3CD, PIK3R1, PIK3R2, PIK3R4, PIK3R5, PIKFYVE, PIN1, PINK1, PIP5K1C, PITPNM3, PITRM1, PITX1, PITX2, PITX3, PKD1, PKD1L1, PKD2, PKHD1, PKLR, PKM, PKP1, PKP2, PLA2G2A, PLA2G4A, PLA2G5, PLA2G6, PLA2G7, PLAA, PLAG1, PLAT, PLB1, PLCB1, PLCB4, PLCD1, PLCE1, PLCG2, PLCZ1, PLD1, PLD3, PLEC, PLEKHA5, PLEKHA7, PLEKHG2, PLEKHG4, PLEKHG5, PLEKHM1, PLEKHM2, PLG, PLK4, PLN, PLOD1, PLOD2, PLOD3, PLP1, PLS3, PLVAP, PLXNA4, PLXNB1, PLXND1, PMFBP1, PMM2, PMP2, PMP22, PMPCA, PMPCB, PMS1, PMS2, PMVK, PNKD, PNKP, PNLIP, PNP, PNPLA1, PNPLA2, PNPLA4, PNPLA6, PNPLA8, PNPO, PNPT1, POC1A, POC1B, POC5, PODXL, POFUT1, POGLUT1, POGZ, POLA1, POLD1, POLE, POLE2, POLG, POLG2, POLH, POLQ, POLR1A, POLR1C, POLR1D, POLR2C, POLR3A, POLR3B, POLR3K, POMC, POMGNT1, POMGNT2, POMK, POMP, POMT1, POMT2, PON1,

PON2, PON3, POP1, POR, PORCN, POT1, POU1F1, POU3F4, POU4F3, POU5F1, PPA2, PPARG, PPCS, PPIB, PPIP5K2, PPM1D, PPM1K, PPOX, PPP1CB, PPP1R12B, PPP1R13L, PPP1R15B, PPP1R21, PPP1R3A, PPP1R3C, PPP2CA, PPP2R1A, PPP2R2B, PPP2R5B, PPP2R5C, PPP2R5D, PPP3CA, PPT1, PQBP1, PRB3, PRCD, PRDM12, PRDM13, PRDM16, PRDM5, PRDM6, PRDM8, PRDX1, PREPL, PREX1, PREX2, PRF1, PRG4, PRICKLE1, PRICKLE2, PRICKLE3, PRIMA1, PRIMPOL, PRKAA1, PRKACG, PRKAG2, PRKAG3, PRKAR1A, PRKAR1B, PRKCA, PRKCB, PRKCD, PRKCE, PRKCG, PRKCSH, PRKD1, PRKDC, PRKG1, PRKRA, PRLR, PRM2, PRMT7, PRMT9, PRND, PRNP, PROC, PROCR, PRODH, PRODH2, PROK2, PROKR2, PROM1, PROP1, PROS1, PROZ, PRPF3, PRPF31, PRPF4, PRPF6, PRPF8, PRPH, PRPH2, PRPS1, PRR12, PRRT2, PRRX1, PRSS1, PRSS12, PRSS56, PRUNE1, PRX, PSAP, PSAT1, PSEN1, PSEN2, PSENEN, PSMA3, PSMB4, PSMB8, PSMB9, PSMC3IP, PSMD12, PSPH, PSPN, PSTPIP1, PTCH1, PTCH2, PTCHD1, PTDSS1, PTEN, PTF1A, PTGIS, PTGS1, PTH, PTH1R, PTHLH, PTK7, PTPN11, PTPN14, PTPN22, PTPN23, PTPN4, PTPRC, PTPRD, PTPRF, PTPRO, PTPRQ, PTRF, PTRH2, PTS, PUF60, PUM1, PURA, PUS1, PUS3, PUS7, PXDN, PYCR1, PYCR2, PYGL, PYGM, PYHIN1, PYROXD1, QARS, QDPR, QKI, QRICH1, QRICH2, QRSL1, RAB10, RAB11A, RAB11B, RAB12, RAB18, RAB23, RAB27A, RAB28, RAB2A, RAB33B, RAB39B, RAB3GAP1, RAB3GAP2, RAB43, RAB7A, RABL6, RAC1, RAC2, RAD18, RAD21, RAD50, RAD51, RAD51B, RAD51C, RAD51D, RAD52, RAD54B, RAD54L, RAF1, RAG1, RAG2, RAI1, RALA, RALGDS, RANBP17, RANBP2, RANGRF, RAP1B, RAPGEF2, RAPSN, RARB, RARS, RARS2, RASA1, RASA2, RASAL1, RASGRP1, RASGRP2, RAX, RAX2, RB1, RBBP6, RBBP8, RBCK1, RBFOX1, RBFOX3, RBL1, RBM10, RBM12, RBM20, RBM27, RBM28, RBM7, RBM8A, RBP1, RBP3, RBP4, RBPJ, RBSN, RCBTB1, RCC1, RD3, RDH11, RDH12, RDH5, RDX, REC114, RECQL, RECQL4, RECQL5, REEP1, REEP2, REEP4, REEP6, RELA, RELB, RELN, REN, REPS1, RERE, REST, RET, REV3L, RFC1, RFT1, RFX5, RFX6, RFXANK, RFXAP, RGR, RGS2, RGS6, RGS7, RGS9, RGS9BP, RHAG, RHBDD2, RHBDF2, RHCE, RHEB, RHNO1, RHO, RHOBTB2, RHOH, RHOXF2, RIC3, RIMS1, RIN1, RIN2, RING1, RIPK1, RIPK2, RIPK4, RIPPLY1, RIPPLY2, RIT1, RLBP1, RLIM, RMI1, RMND1, RMRP, RNASEH1, RNASEH2A, RNASEH2B, RNASEH2C, RNASEL, RNASET2, RNF113A, RNF114, RNF125, RNF13, RNF135, RNF168, RNF170, RNF20, RNF213, RNF216, RNF31, RNF43, RNLS, RNPC3, RNU4ATAC, ROBO1, ROBO2, ROBO3, ROBO4, ROGDI, ROM1, ROR1, ROR2, RORA, RORB, RORC, RP1, RP1L1, RP2, RP9, RPE65, RPGR, RPGRIP1, RPGRIP1L, RPH3A, RPIA, RPL10, RPL11, RPL15, RPL18, RPL19, RPL21, RPL26, RPL27, RPL31, RPL35A, RPL4, RPL5, RPL9, RPLP0, RPS10, RPS15A, RPS17, RPS19, RPS20, RPS23, RPS24, RPS26, RPS27, RPS28, RPS29, RPS6KA3, RPS7, RPSA, RRAGA, RRAGC, RRAS, RRM2B, RRP8, RS1, RSPH1, RSPH3, RSPH4A, RSPH9, RSPO1, RSPO4, RSPRY1, RSRC1, RTEL1, RTN2, RTN4IP1, RTTN, RUBCN, RUNDC1, RUNX1, RUNX2, RUSC2, RXRB, RYK, RYR1, RYR2, RYR3, S1PR2, S1PR3, SAA4, SACS, SAE1, SAG, SALL1, SALL2, SALL4, SAMD11, SAMD9, SAMD9L, SAMHD1, SAR1B, SARDH, SARS, SARS2, SART3, SASH1, SASS6, SATB2, SBDS, SBF1, SBF2, SC5D, SCAPER, SCARB1, SCARB2, SCARF2, SCG2, SCHIP1, SCLT1, SCN10A, SCN11A, SCN1A, SCN1B, SCN2A, SCN2B, SCN3A, SCN3B, SCN4A, SCN4B, SCN5A, SCN8A, SCN9A, SCNM1, SCNN1A, SCNN1B, SCNN1G, SCO1, SCO2, SCP2, SCRIB, SCYL1, SDCCAG8, SDHA, SDHAF1, SDHAF2, SDHB, SDHC, SDHD, SDR9C7, SEC23A, SEC23B, SEC23IP, SEC24B, SEC24D, SEC61A1, SEC63, SECISBP2, SELENBP1, SEMA3A, SEMA3C, SEMA3D, SEMA3E, SEMA4A, SEMA5A, SEPSECS, SEPT12, SEPT9, SERAC1, SERPINA1,

SERPINA3, SERPINA6, SERPINA7, SERPINB4, SERPINB6, SERPINB7, SERPINB8, SERPINC1, SERPIND1, SERPINE1, SERPINF1, SERPINF2, SERPING1, SERPINH1, SERPINI1, SET, SETBP1, SETD1A, SETD1B, SETD2, SETD5, SETD6, SETX, SF3B4, SFRP4, SFTPA1, SFTPA2, SFTPB, SFTPC, SFXN4, SGCA, SGCB, SGCD, SGCE, SGCG, SGO1, SGO2, SGPL1, SGSH, SH2B1, SH2B3, SH2D1A, SH3BP2, SH3GL1, SH3PXD2B, SH3TC2, SHANK1, SHANK2, SHANK3, SHBG, SHH, SHOC2, SHOX, SHOX2, SHPK, SHROOM3, SHROOM4, SI, SIAE, SIGIRR, SIGLEC1, SIGMAR1, SIK1, SIK3, SIL1, SIM1, SIN3A, SIPA1L1, SIPA1L3, SIRT1, SIRT3, SIRT6, SIX1, SIX2, SIX3, SIX5, SIX6, SKI, SKIV2L, SLBP, SLC10A1, SLC10A2, SLC10A7, SLC11A2, SLC12A1, SLC12A2, SLC12A3, SLC12A5, SLC12A6, SLC13A5, SLC14A1, SLC16A1, SLC16A12, SLC16A2, SLC17A3, SLC17A5, SLC17A8, SLC17A9, SLC18A2, SLC18A3, SLC19A2, SLC19A3, SLC1A1, SLC1A2, SLC1A3, SLC1A4, SLC20A2, SLC22A12, SLC22A4, SLC22A5, SLC24A1, SLC24A4, SLC24A5, SLC25A1, SLC25A10, SLC25A11, SLC25A12, SLC25A13, SLC25A15, SLC25A19, SLC25A20, SLC25A21, SLC25A22, SLC25A24, SLC25A26, SLC25A3, SLC25A32, SLC25A38, SLC25A4, SLC25A40, SLC25A42, SLC25A46, SLC26A1, SLC26A2, SLC26A3, SLC26A4, SLC26A5, SLC26A8, SLC27A1, SLC27A4, SLC27A5, SLC28A1, SLC29A1, SLC29A2, SLC29A3, SLC29A4, SLC2A1, SLC2A10, SLC2A2, SLC2A9, SLC30A10, SLC30A2, SLC30A9, SLC31A1, SLC33A1, SLC34A1, SLC34A2, SLC34A3, SLC35A1, SLC35A2, SLC35A3, SLC35B1, SLC35C1, SLC35D1, SLC35D3, SLC36A2, SLC37A4, SLC38A10, SLC38A8, SLC39A13, SLC39A14, SLC39A4, SLC39A5, SLC39A8, SLC3A1, SLC40A1, SLC41A1, SLC44A1, SLC45A1, SLC45A2, SLC46A1, SLC4A1, SLC4A11, SLC4A3, SLC4A4, SLC51B, SLC52A1, SLC52A2, SLC52A3, SLC5A1, SLC5A2, SLC5A5, SLC5A6, SLC5A7, SLC6A1, SLC6A17, SLC6A19, SLC6A2, SLC6A3, SLC6A5, SLC6A8, SLC6A9, SLC7A14, SLC7A2, SLC7A3, SLC7A5, SLC7A7, SLC7A8, SLC7A9, SLC9A1, SLC9A3, SLC9A3R1, SLC9A6, SLC9A9, SLCO1B1, SLCO1B3, SLCO2A1, SLFN14, SLIT2, SLITRK1, SLITRK5, SLITRK6, SLMAP, SLURP1, SLX4, SMAD1, SMAD2, SMAD3, SMAD4, SMAD6, SMAD9, SMARCA2, SMARCA4, SMARCAL1, SMARCB1, SMARCC1, SMARCC2, SMARCE1, SMC1A, SMC3, SMCHD1, SMG9, SMN1, SMN2, SMO, SMOC1, SMOC2, SMPD1, SMPX, SMS, SMURF2, SMYD1, SNAI2, SNAP25, SNAP29, SNCA, SNCB, SNIP1, SNORD118, SNRNP200, SNRPA, SNRPE, SNTA1, SNX10, SNX14, SNX27, SNX3, SOBP, SOCS3, SOCS4, SOD1, SOHLH1, SON, SORBS3, SORCS3, SORL1, SORT1, SOS1, SOS2, SOST, SOX10, SOX11, SOX17, SOX18, SOX2, SOX3, SOX5, SOX8, SOX9, SP110, SP2, SP7, SPAG1, SPAG17, SPARC, SPARCL1, SPAST, SPATA16, SPATA17, SPATA5, SPATA7, SPATC1L, SPECC1L, SPEG, SPG11, SPG21, SPG7, SPI1, SPIDR, SPINK1, SPINK2, SPINK5, SPINT2, SPOCK1, SPOP, SPP1, SPP2, SPPL2A, SPR, SPRED1, SPRTN, SPRY2, SPTA1, SPTAN1, SPTB, SPTBN2, SPTBN4, SPTBN5, SPTLC1, SPTLC2, SQSTM1, SRA1, SRC, SRCAP, SRD5A2, SRD5A3, SREBF1, SRGAP1, SRGAP3, SRI, SRP72, SRPK2, SRPK3, SRPX2, SRRM2, SRSF11, SRY, SS18L1, SSH1, SSR4, SSTR5, SSUH2, ST14, ST3GAL3, ST3GAL5, ST5, ST6GALNAC5, ST7, STAC3, STAG1, STAG2, STAG3, STAMBP, STAP1, STAR, STARD8, STARD9, STAT1, STAT2, STAT3, STAT4, STAT5B, STIL, STIM1, STK11, STK36, STK4, STON1, STRA6, STRA8, STRADA, STRC, STS, STT3A, STT3B, STUB1, STX11, STX1A, STX1B, STX2, STX3, STX7, STXBP1, STXBP2, STXBP5, STXBP5L, STYXL1, SUCLA2, SUCLG1, SUCO, SUFU, SUGCT, SULT2B1, SUMF1, SUMO1, SUN1, SUN2, SUN5, SUOX, SUPT5H, SURF1, SUZ12, SV2A, SV2C, SYCE1, SYCP3, SYN1, SYN2, SYNE1, SYNE4, SYNGAP1, SYNJ1, SYNM, SYNPO, SYP, SYT1, SYT14, SYT2, SZT2, T, TAB2, TAC3, TACC2, TACO1, TACR3, TACSTD2, TADA2A, TAF1, TAF13, TAF15, TAF1A, TAF2, TAF4B, TAF6, TAF8, TALDO1,

TANC2, TANGO2, TAOK2, TAP1, TAP2, TAPBP, TAPT1, TARDBP, TARS2, TAT, TAX1BP3, TAZ, TBC1D1, TBC1D20, TBC1D23, TBC1D24, TBC1D31, TBC1D32, TBC1D4, TBC1D7, TBCD, TBCE, TBCK, TBK1, TBL1X, TBL1XR1, TBR1, TBRG1, TBX1, TBX15, TBX18, TBX19, TBX2, TBX20, TBX22, TBX3, TBX4, TBX5, TBX6, TBXA2R, TBXAS1, TCAP, TCF12, TCF20, TCF3, TCF4, TCF7L2, TCHH, TCIRG1, TCN1, TCN2, TCOF1, TCTEX1D2, TCTN1, TCTN2, TCTN3, TDGF1, TDO2, TDP1, TDP2, TDRD6, TDRD7, TDRD9, TEAD1, TECPR2, TECR, TECRL, TECTA, TEK, TEKT1, TELO2, TENM1, TENM3, TENM4, TERC, TERF2, TERF2IP, TERT, TET2, TEX11, TEX14, TEX15, TF, TFAM, TFAP2A, TFAP2B, TFB2M, TFG, TFR2, TFRC, TG, TGDS, TGFB1, TGFB2, TGFB3, TGFBI, TGFBR1, TGFBR2, TGFBR3, TGIF1, TGM1, TGM2, TGM3, TGM5, TGM6, TH, THAP1, THAP11, THBD, THBS1, THG1L, THOC2, THOC6, THPO, THRA, THRB, THSD1, THUMPD1, TIA1, TICAM1, TIMM22, TIMM44, TIMM50, TIMM8A, TIMP3, TINF2, TIRAP, TJP2, TK2, TKT, TLE1, TLE6, TLK2, TLL1, TLN2, TLR3, TLR9, TM4SF20, TM6SF2, TMC1, TMC6, TMC8, TMCO1, TMEM106B, TMEM107, TMEM114, TMEM126A, TMEM126B, TMEM127, TMEM132E, TMEM135, TMEM138, TMEM165, TMEM173, TMEM199, TMEM216, TMEM230, TMEM231, TMEM237, TMEM240, TMEM260, TMEM38B, TMEM43, TMEM5, TMEM65, TMEM67, TMEM70, TMEM92, TMEM94, TMEM98, TMIE, TMLHE, TMPO, TMPRSS15, TMPRSS3, TMPRSS4, TMPRSS5, TMPRSS6, TMTC3, TMX3, TNC, TNFAIP3, TNFRSF10B, TNFRSF11A, TNFRSF11B, TNFRSF13B, TNFRSF1A, TNFRSF4, TNFRSF6B, TNFSF11, TNFSF12, TNFSF8, TNIK, TNNC1, TNNI2, TNNI3, TNNI3K, TNNT1, TNNT2, TNNT3, TNPO3, TNRC6B, TNS2, TNXB, TOE1, TONSL, TOP2B, TOP3A, TOPORS, TOR1A, TOR1AIP1, TP53, TP53BP2, TP53RK, TP63, TPH1, TPH2, TPI1, TPK1, TPM1, TPM2, TPM3, TPM4, TPMT, TPO, TPP1, TPP2, TPRKB, TPRN, TRAF3, TRAF3IP1, TRAF3IP2, TRAF6, TRAIP, TRAK1, TRAP1, TRAPPC11, TRAPPC12, TRAPPC2, TRAPPC2L, TRAPPC6A, TRAPPC6B, TRAPPC9, TRDN, TREM2, TREX1, TRHR, TRIM2, TRIM22, TRIM28, TRIM32, TRIM36, TRIM37, TRIM44, TRIM54, TRIM55, TRIM63, TRIM8, TRIO, TRIOBP, TRIP11, TRIP12, TRIP13, TRIP4, TRIT1, TRMT1, TRMT10A, TRMT10C, TRMT5, TRMU, TRNT1, TRPA1, TRPC6, TRPM1, TRPM2, TRPM4, TRPM6, TRPS1, TRPV3, TRPV4, TRPV6, TRRAP, TSC1, TSC2, TSEN15, TSEN2, TSEN34, TSEN54, TSFM, TSHB, TSHR, TSHZ1, TSPAN12, TSPAN7, TSPEAR, TSPYL1, TSR2, TTBK2, TTC19, TTC21B, TTC25, TTC37, TTC7A, TTC8, TTF1, TTF2, TTI1, TTI2, TTLL5, TTN, TTPA, TTR, TUB, TUBA1A, TUBA3D, TUBA3E, TUBA4A, TUBA8, TUBB, TUBB1, TUBB2A, TUBB2B, TUBB3, TUBB4A, TUBB4B, TUBB6, TUBB8, TUBG1, TUBGCP4, TUBGCP6, TUFM, TUFT1, TULP1, TULP4, TUSC3, TWIST1, TWIST2, TXN2, TXNDC15, TXNL4A, TXNRD1, TXNRD2, TYK2, TYMP, TYR, TYROBP, TYRP1, UBA1, UBA3, UBA5, UBA7, UBE2A, UBE2B, UBE2T, UBE3A, UBE3B, UBE3C, UBIAD1, UBN2, UBQLN2, UBQLN4, UBR1, UBR5, UBR7, UBTF, UCHL1, UCP2, UCP3, UFC1, UFM1, UFSP2, UGCG, UGDH, UGGT1, UGT1A1, UHRF1, UMOD, UMPS, UNC119, UNC13A, UNC13D, UNC45A, UNC45B, UNC50, UNC5C, UNC79, UNC80, UNC93B1, UNG, UPB1, UPF3B, UPK3A, UQCC2, UQCC3, UQCRB, UQCRC2, UQCRQ, UROC1, UROD, UROS, USB1, USH1C, USH1G, USH2A, USMG5, USP15, USP18, USP26, USP27X, USP34, USP44, USP45, USP7, USP8, USP9X, USP9Y, USPL1, UVRAG, UVSSA, VAC14, VAMP1, VANGL1, VANGL2, VAPB, VARS, VARS2, VAV1, VAX1, VAX2, VCAN, VCL, VCP, VDR, VEGFA, VEGFC, VHL, VIL1, VIM, VIP, VIPAS39, VKORC1, VLDLR, VMA21, VPS11, VPS13A, VPS13B, VPS13C, VPS13D, VPS16, VPS33A, VPS33B, VPS35, VPS37A, VPS45, VPS4B, VPS53, VRK1, VSIG10L, VSX1, VSX2, VWA2, VWA3B, VWF, WAC, WARS, WARS2, WAS, WBP2, WDFY3, WDPCP,

WDR1, WDR11, WDR13, WDR19, WDR26, WDR34, WDR35, WDR36, WDR4, WDR45, WDR45B, WDR48, WDR5, WDR60, WDR62, WDR66, WDR72, WDR73, WDR81, WDR87, WDR93, WEE2, WFS1, WHRN, WHSC1, WIF1, WIPF1, WIPI2, WISP3, WNK1, WNK4, WNT1, WNT10A, WNT10B, WNT2B, WNT3, WNT3A, WNT4, WNT5A, WNT7A, WNT8A, WNT9B, WRAP53, WRN, WT1, WWOX, XAF1, XDH, XIAP, XIRP1, XIST, XK, XPA, XPC, XPNPEP3, XPO5, XPR1, XRCC1, XRCC2, XRCC3, XRCC4, XYLT1, XYLT2, YAP1, YARS, YARS2, YME1L1, YWHAE, YWHAG, YY1, YY1AP1, ZAP70, ZAR1, ZBTB11, ZBTB16, ZBTB17, ZBTB18, ZBTB20, ZBTB24, ZBTB33, ZBTB40, ZBTB42, ZC3H14, ZC3H4, ZC4H2, ZCCHC12, ZCCHC8, ZDBF2, ZDHHC15, ZDHHC9, ZEB1, ZEB2, ZFHX2, ZFP36L1, ZFP57, ZFPM2, ZFR, ZFYVE16, ZFYVE26, ZFYVE27, ZHX3, ZIC1, ZIC2, ZIC3, ZMPSTE24, ZMYM3, ZMYM6, ZMYND10, ZMYND11, ZMYND15, ZNF141, ZNF143, ZNF148, ZNF335, ZNF34, ZNF341, ZNF407, ZNF408, ZNF41, ZNF423, ZNF462, ZNF469, ZNF513, ZNF526, ZNF543, ZNF589, ZNF592, ZNF599, ZNF644, ZNF674, ZNF687, ZNF711, ZNF750, ZNF804A, ZNF81, ZNHIT3, ZP1, ZP2, ZP3, ZPBP, ZPR1, ZSWIM6

Supplementary Table 2. Demographics of patients with trio test.

Trio#	Sex	Age (Months)	NDD with epilepsy	Panel	Trio method
1	Male	13	Yes	Epilepsy	Single gene
2	Female	48	Yes	NDD	NGS-based
3	Female	7	No	NDD	Single gene
4	Female	156	Yes	NDD	Single gene
5	Female	24	No	NDD	Single gene
6	Female	8	No	NDD	NGS-based
7	Male	28	Yes	NDD	NGS-based
8	Male	21	Yes	Epilepsy	Single gene
9	Male	222	Yes	NDD	Single gene
10	Male	46	Yes	NDD	NGS-based
11	Female	19	Yes	Epilepsy	Single gene
12	Male	49	No	NDD	Single gene
13	Male	39	Yes	Epilepsy	Single gene
14	Male	60	No	NDD	Single gene
15	Female	109	Yes	NDD	NGS-based
16	Male	22	Yes	Epilepsy	Single gene
17	Male	42	Yes	Epilepsy	Single gene
18	Male	5	Yes	Epilepsy	Single gene
19	Female	7	Yes	Epilepsy	Single gene
20	Male	19	Yes	Epilepsy	Single gene
21	Male	41	Yes	Epilepsy	Single gene
22	Male	83	Yes	Epilepsy	Single gene
23	Male	21	Yes	Epilepsy	Single gene
24	Male	211	Yes	Epilepsy	Single gene
25	Female	65	Yes	Epilepsy	Single gene
26	Female	5	Yes	Epilepsy	Single gene
27	Female	14	Yes	Epilepsy	Single gene
28	Male	22	Yes	Epilepsy	Single gene
29	Male	38	Yes	Epilepsy	Single gene

30	Female	78	Yes	Epilepsy	Single gene
31	Male	36	Yes	NDD	Single gene
32	Male	100	Yes	NDD	Single gene
33	Male	44	No	NDD	Single gene
34	Female	126	Yes	NDD	Single gene
35	Female	21	No	NDD	Single gene
36	Male	18	Yes	Epilepsy	Single gene
37	Female	22	No	NDD	Single gene
38	Male	167	Yes	NDD	Single gene
39	Male	1	No	NDD	Single gene
40	Female	40	No	NDD	Single gene
41	Male	107	Yes	Epilepsy	Single gene
42	Male	10	Yes	NDD	Single gene
43	Male	93	Yes	NDD	Single gene
44	Male	76	No	NDD	Single gene
45	Female	40	Yes	NDD	Single gene
46	Female	86	Yes	NDD	Single gene
47	Male	110	Yes	NDD	Single gene
48	Female	16	No	NDD	Single gene
49	Male	20	No	NDD	Single gene
50	Male	191	No	NDD	Single gene
51	Female	181	Yes	NDD	Single gene
52	Female	39	No	NDD	NGS-based
53	Male	28	No	MCD	Single gene
54	Female	17	Yes	NDD	Single gene
55	Male	4	Yes	Epilepsy	Single gene
56	Male	6	Yes	Epilepsy	Single gene
57	Female	164	Yes	NDD	NGS-based
58	Male	2	Yes	Epilepsy	Single gene

59	Female	55	Yes	Epilepsy	Single gene
60	Female	7	Yes	Epilepsy	Single gene
61	Male	56	No	NDD	NGS-based
62	Male	114	Yes	NDD	Single gene
63	Male	32	Yes	NDD	Single gene
64	Male	12	No	NDD	Single gene
65	Male	30	No	NDD	Single gene
66	Male	8	Yes	Epilepsy	Single gene
67	Female	45	Yes	NDD	Single gene
68	Male	65	Yes	Epilepsy	Single gene
69	Female	6	Yes	Epilepsy	Single gene
70	Male	62	No	NDD	Single gene
71	Male	82	Yes	Epilepsy	Single gene
72	Female	31	Yes	NDD	Single gene
73	Female	10	Yes	NDD	Single gene
74	Female	130	No	NDD	Single gene
75	Female	120	Yes	NDD	Single gene
76	Female	139	No	MCD	Single gene
77	Female	99	No	NDD	Single gene
78	Female	2	Yes	Epilepsy	Single gene
79	Female	153	Yes	Epilepsy	Single gene
80	Female	6	Yes	Epilepsy	Single gene
81	Male	6	Yes	Epilepsy	Single gene
82	Male	66	Yes	NDD	Single gene
83	Female	111	Yes	NDD	Single gene
84	Male	145	Yes	NDD	Single gene
85	Male	32	Yes	Epilepsy	Single gene
86	Female	203	Yes	Epilepsy	Single gene
87	Male	26	Yes	Epilepsy	Single gene
88	Female	69	No	NDD	Single gene
89	Male	306	Yes	Epilepsy	Single gene
90	Female	35	Yes	Epilepsy	Single gene
91	Male	126	No	NDD	Single gene

92	Male	109	Yes	NDD	Single gene
93	Female	83	Yes	NDD	Single gene
94	Male	98	Yes	Epilepsy	Single gene
95	Male	6	Yes	Epilepsy	Single gene
96	Female	66	Yes	Epilepsy	Single gene
97	Male	6	Yes	Epilepsy	Single gene
98	Male	208	Yes	Epilepsy	Single gene
99	Male	93	Yes	Epilepsy	Single gene
100	Female	81	Yes	Epilepsy	Single gene
101	Male	8	Yes	NDD	Single gene
102	Male	29	Yes	Epilepsy	Single gene
103	Female	8	Yes	NDD	Single gene
104	Male	8	Yes	Epilepsy	Single gene
105	Male	13	Yes	MCD	Single gene
106	Male	73	Yes	Epilepsy	Single gene
107	Male	28	Yes	NDD	Single gene
108	Male	71	Yes	NDD	NGS-based
109	Male	16	Yes	Epilepsy	Single gene
110	Male	8	Yes	Epilepsy	Single gene
111	Male	213	No	NDD	Single gene
112	Male	13	No	NDD	Single gene
113	Male	252	Yes	NDD	NGS-based
114	Male	138	No	NDD	NGS-based
115	Female	108	No	NDD	Single gene
116	Female	15	Yes	NDD	Single gene
117	Female	22	Yes	Epilepsy	Single gene
118	Female	26	Yes	NDD	Single gene
119	Male	316	No	NDD	Single gene
120	Male	45	No	NDD	Single gene
121	Male	60	No	NDD	NGS-based
122	Male	80	Yes	NDD	Single gene
123	Male	37	No	NDD	Single gene

124	Male	110	Yes	NDD	NGS-based
125	Male	11	Yes	Epilepsy	Single gene
126	Male	87	Yes	Epilepsy	Single gene
127	Female	7	Yes	Epilepsy	Single gene
128	Male	93	Yes	Epilepsy	Single gene
129	Male	46	Yes	NDD	Single gene
130	Male	21	Yes	NDD	NGS-based
131	Female	104	Yes	Epilepsy	Single gene
132	Male	61	No	NDD	Single gene
133	Male	97	Yes	NDD	Single gene
134	Female	25	No	NDD	NGS-based
135	Male	33	Yes	NDD	Single gene
136	Male	244	Yes	Epilepsy	Single gene
137	Male	21	Yes	Epilepsy	Single gene
138	Male	5	Yes	Epilepsy	Single gene
139	Male	45	Yes	NDD	Single gene
140	Male	167	No	MCD	Single gene
141	Female	67	No	NDD	Single gene
142	Female	21	No	NDD	NGS-based
143	Male	130	Yes	NDD	Single gene
144	Female	75	No	NDD	Single gene
145	Female	72	No	NDD	Single gene
146	Male	40	No	NDD	Single gene
147	Female	219	Yes	Epilepsy	Single gene
148	Male	13	Yes	Epilepsy	Single gene
149	Female	81	Yes	NDD	Single gene
150	Male	61	Yes	Epilepsy	Single gene
151	Female	39	Yes	Epilepsy	Single gene
152	Female	78	Yes	NDD	Single gene
153	Male	26	Yes	Epilepsy	Single gene
154	Female	62	Yes	Epilepsy	Single gene
155	Male	55	Yes	NDD	NGS-based
156	Female	57	Yes	Epilepsy	Single gene

157	Male	59	No	NDD	NGS-based
158	Female	10	No	NDD	Single gene
159	Female	67	Yes	NDD	Single gene
160	Male	322	No	NDD	Single gene
161	Male	150	Yes	NDD	Single gene
162	Male	109	Yes	NDD	Single gene
163	Male	80	Yes	NDD	Single gene
164	Male	305	Yes	NDD	Single gene
165	Male	34	Yes	Epilepsy	Single gene
166	Male	77	Yes	Epilepsy	Single gene
167	Female	146	Yes	Epilepsy	Single gene
168	Male	7	Yes	NDD	Single gene
169	Male	19	No	NDD	NGS-based
170	Female	15	No	NDD	NGS-based
171	Male	64	Yes	Epilepsy	Single gene
172	Female	41	Yes	NDD	Single gene
173	Female	103	No	Epilepsy	Single gene
174	Male	24	Yes	Epilepsy	Single gene
175	Female	68	Yes	NDD	NGS-based
176	Male	84	Yes	NDD	Single gene
177	Male	78	Yes	NDD	Single gene
178	Female	11	Yes	Epilepsy	Single gene
179	Female	8	Yes	NDD	Single gene
180	Female	303	Yes	NDD	Single gene
181	Female	7	Yes	Epilepsy	Single gene
182	Male	36	Yes	Epilepsy	Single gene
183	Male	50	No	NDD	Single gene
184	Female	11	No	NDD	Single gene
185	Male	126	Yes	Epilepsy	Single gene
186	Male	64	Yes	NDD	Single gene
187	Male	40	Yes	Epilepsy	Single gene

188	Female	27	Yes	NDD	Single gene
189	Male	197	Yes	NDD	Single gene
190	Female	12	No	NDD	Single gene
191	Male	91	Yes	NDD	Single gene
192	Male	62	No	NDD	Single gene
193	Male	22	No	NDD	Single gene
194	Female	120	Yes	NDD	NGS-based
195	Female	16	Yes	NDD	NGS-based
196	Male	24	Yes	NDD	NGS-based
197	Female	47	No	NDD	NGS-based
198	Male	10	Yes	Epilepsy	Single gene
199	Male	45	No	NDD	Single gene
200	Male	41	Yes	Epilepsy	Single gene
201	Female	6	Yes	Epilepsy	Single gene
202	Male	34	Yes	NDD	Single gene
203	Female	57	Yes	Epilepsy	Single gene
204	Male	14	Yes	NDD	NGS-based
205	Female	19	Yes	NDD	Single gene
206	Male	21	No	NDD	Single gene
207	Female	178	Yes	NDD	NGS-based
208	Female	137	Yes	Epilepsy	NGS-based
209	Male	3	Yes	Epilepsy	Single gene
210	Female	6	Yes	NDD	Single gene
211	Female	26	Yes	Epilepsy	Single gene
212	Male	33	Yes	Epilepsy	Single gene
213	Male	61	Yes	Epilepsy	Single gene
214	Male	133	Yes	NDD	NGS-based
215	Female	15	Yes	Epilepsy	Single gene
216	Male	151	Yes	Epilepsy	Single gene
217	Male	52	Yes	Epilepsy	Single gene

218	Male	142	Yes	Epilepsy	Single gene
219	Male	26	Yes	Epilepsy	Single gene
220	Male	105	Yes	Epilepsy	Single gene
221	Male	118	Yes	Epilepsy	Single gene
222	Female	293	Yes	Epilepsy	Single gene
223	Male	16	Yes	Epilepsy	Single gene
224	Female	32	Yes	NDD	NGS-based
225	Female	322	Yes	Epilepsy	Single gene
226	Male	258	Yes	Epilepsy	Single gene
227	Male	29	Yes	Epilepsy	Single gene
228	Male	2	Yes	Epilepsy	Single gene
229	Male	132	Yes	NDD	NGS-based
230	Female	93	Yes	Epilepsy	NGS-based
231	Male	5	Yes	NDD	NGS-based
232	Male	1	Yes	Epilepsy	NGS-based
233	Male	7	Yes	Epilepsy	Single gene
234	Female	82	Yes	NDD	Single gene
235	Female	4	Yes	NDD	Single gene
236	Male	57	Yes	Epilepsy	Single gene
237	Female	6	Yes	Epilepsy	Single gene
238	Male	2	Yes	Epilepsy	Single gene
239	Female	61	Yes	Epilepsy	Single gene
240	Female	13	Yes	Epilepsy	Single gene
241	Female	153	Yes	Epilepsy	Single gene
242	Female	40	Yes	Epilepsy	Single gene
243	Male	11	Yes	NDD	Single gene
244	Male	93	Yes	NDD	Single gene
245	Female	130	Yes	NDD	Single gene
246	Female	8	Yes	NDD	Single gene
247	Female	45	No	NDD	Single gene
248	Female	47	Yes	NDD	Single gene
249	Female	17	Yes	NDD	Single gene
250	Female	195	Yes	Epilepsy	Single gene

251	Female	133	No	NDD	Single gene
252	Male	128	Yes	NDD	Single gene
253	Male	17	No	NDD	Single gene
254	Male	7	No	NDD	Single gene
255	Male	7	No	NDD	Single gene
256	Female	24	No	MCD	Single gene
257	Female	94	Yes	NDD	Single gene
258	Male	40	No	NDD	Single gene
259	Female	25	No	NDD	NGS-based
260	Female	49	No	NDD	Single gene
261	Female	98	Yes	NDD	Single gene
262	Male	57	No	NDD	Single gene
263	Female	52	No	NDD	Single gene
264	Male	8	Yes	Epilepsy	Single gene
265	Female	102	No	NDD	Single gene
266	Male	96	Yes	NDD	Single gene
267	Female	49	No	NDD	Single gene
268	Female	95	Yes	NDD	Single gene
269	Female	1	Yes	NDD	Single gene
270	Male	194	No	NDD	Single gene
271	Female	34	No	NDD	Single gene
272	Female	15	No	NDD	Single gene
273	Male	8	No	NDD	Single gene
274	Female	12	Yes	NDD	Single gene
275	Male	39	No	NDD	Single gene
276	Female	21	Yes	NDD	Single gene
277	Male	38	Yes	NDD	NGS-based
278	Male	200	No	NDD	Single gene
279	Female	6	Yes	NDD	Single gene

280	Male	19	Yes	Epilepsy	Single gene
281	Male	79	Yes	NDD	Single gene
282	Male	19	No	NDD	Single gene
283	Male	58	Yes	MCD	Single gene
284	Female	56	Yes	NDD	NGS-based
285	Female	37	No	NDD	Single gene
286	Female	65	No	NDD	Single gene
287	Male	63	No	NDD	Single gene
288	Male	34	Yes	NDD	Single gene
289	Female	62	Yes	NDD	Single gene
290	Female	73	No	Epilepsy	Single gene
291	Male	74	Yes	NDD	NGS-based
292	Male	77	No	NDD	Single gene
293	Male	7	No	NDD	Single gene
294	Female	3	Yes	NDD	Single gene
295	Male	57	Yes	Epilepsy	Single gene
296	Female	83	Yes	Epilepsy	Single gene
297	Female	101	No	NDD	Single gene
298	Female	5	Yes	Epilepsy	Single gene
299	Female	13	Yes	Epilepsy	Single gene
300	Female	42	Yes	Epilepsy	Single gene
301	Female	71	Yes	Epilepsy	Single gene
302	Male	111	No	NDD	Single gene
303	Male	64	No	NDD	Single gene
304	Female	48	Yes	NDD	Single gene
305	Male	32	Yes	NDD	Single gene
306	Male	125	Yes	NDD	Single gene
307	Female	201	No	NDD	Single gene
308	Female	2	Yes	Epilepsy	Single gene
309	Male	25	Yes	NDD	Single gene

310	Female	51	Yes	NDD	Single gene
311	Female	9	Yes	Epilepsy	Single gene
312	Female	109	Yes	NDD	Single gene
313	Male	41	Yes	Epilepsy	Single gene
314	Male	9	Yes	NDD	Single gene
315	Male	64	No	NDD	Single gene
316	Female	75	Yes	NDD	NGS-based
317	Male	72	No	NDD	Single gene
318	Male	2	Yes	NDD	Single gene
319	Male	46	No	NDD	Single gene
320	Female	8	Yes	Epilepsy	Single gene
321	Male	6	Yes	Epilepsy	Single gene
322	Male	57	Yes	Epilepsy	Single gene
323	Female	60	Yes	NDD	Single gene
324	Male	32	No	NDD	NGS-based
325	Male	70	No	MCD	Single gene
326	Female	50	No	NDD	Single gene
327	Female	100	Yes	NDD	NGS-based
328	Female	60	No	NDD	Single gene
329	Male	12	Yes	Epilepsy	Single gene
330	Male	51	Yes	NDD	NGS-based
331	Female	69	No	NDD	Single gene
332	Male	30	No	NDD	Single gene
333	Male	35	Yes	NDD	NGS-based
334	Female	37	No	NDD	Single gene
335	Male	48	Yes	NDD	Single gene
336	Female	54	Yes	NDD	Single gene
337	Male	50	No	NDD	Single gene
338	Male	89	Yes	NDD	Single gene
339	Male	44	Yes	Epilepsy	Single gene
340	Female	3	Yes	NDD	Single gene
341	Male	146	Yes	NDD	NGS-based

342	Male	38	Yes	NDD	Single gene
343	Female	163	Yes	Epilepsy	Single gene
344	Female	44	Yes	Epilepsy	Single gene
345	Male	86	Yes	NDD	Single gene
346	Male	2	Yes	NDD	Single gene
347	Male	77	Yes	Epilepsy	Single gene
348	Male	34	Yes	Epilepsy	Single gene
349	Male	18	Yes	Epilepsy	Single gene
350	Female	23	Yes	Epilepsy	Single gene
351	Male	27	Yes	NDD	NGS-based
352	Male	84	Yes	Epilepsy	Single gene
353	Male	8	Yes	Epilepsy	Single gene
354	Male	2	Yes	Epilepsy	Single gene
355	Male	32	Yes	Epilepsy	Single gene
356	Male	53	Yes	NDD	Single gene
357	Female	43	Yes	NDD	Single gene
358	Female	259	Yes	NDD	Single gene
359	Female	75	Yes	Epilepsy	Single gene
360	Male	25	Yes	Epilepsy	Single gene
361	Male	20	No	NDD	NGS-based
362	Male	27	Yes	NDD	Single gene
363	Male	10	Yes	Epilepsy	Single gene
364	Female	160	No	NDD	Single gene
365	Male	85	Yes	NDD	Single gene
366	Male	131	Yes	NDD	Single gene
367	Female	40	No	NDD	Single gene
368	Female	3	Yes	Epilepsy	Single gene
369	Male	5	Yes	Epilepsy	Single gene
370	Male	3	Yes	Epilepsy	Single gene
371	Male	14	Yes	NDD	Single gene
372	Male	3	Yes	NDD	Single gene
373	Male	171	Yes	Epilepsy	Single gene

374	Female	72	Yes	NDD	Single gene
375	Female	6	Yes	Epilepsy	Single gene
376	Male	6	Yes	Epilepsy	Single gene
377	Male	5	Yes	Epilepsy	Single gene
378	Female	251	Yes	Epilepsy	Single gene
379	Male	150	Yes	Epilepsy	Single gene
380	Female	35	No	NDD	Single gene
381	Female	4	Yes	NDD	Single gene
382	Female	9	Yes	NDD	Single gene
383	Male	7	Yes	NDD	Single gene
384	Female	78	Yes	NDD	Single gene
385	Female	66	Yes	Epilepsy	Single gene
386	Male	56	Yes	Epilepsy	Single gene
387	Female	13	Yes	Epilepsy	Single gene
388	Female	82	No	NDD	Single gene
389	Male	50	Yes	Epilepsy	Single gene
390	Female	29	Yes	Epilepsy	Single gene
391	Male	37	Yes	Epilepsy	Single gene
392	Male	40	Yes	NDD	Single gene
393	Male	7	Yes	Epilepsy	Single gene
394	Female	40	No	NDD	Single gene
395	Female	159	Yes	Epilepsy	Single gene
396	Male	31	Yes	Epilepsy	Single gene
397	Female	234	Yes	Epilepsy	Single gene
398	Male	171	Yes	Epilepsy	Single gene
399	Male	2	Yes	NDD	Single gene
400	Male	54	No	NDD	Single gene
401	Male	63	No	NDD	Single gene
402	Male	8	Yes	Epilepsy	Single gene
403	Male	261	Yes	Epilepsy	Single gene
404	Male	211	No	NDD	Single gene
405	Male	24	Yes	Epilepsy	Single gene
406	Male	26	Yes	Epilepsy	Single gene

407	Male	104	Yes	NDD	NGS-based
408	Female	33	No	NDD	Single gene
409	Female	156	Yes	NDD	Single gene
410	Male	204	No	NDD	Single gene
411	Male	202	Yes	NDD	NGS-based
412	Female	111	Yes	NDD	Single gene
413	Male	110	No	NDD	Single gene
414	Male	27	No	NDD	Single gene
415	Male	18	No	NDD	NGS-based
416	Female	5	No	NDD	Single gene
417	Male	155	Yes	Epilepsy	Single gene
418	Female	61	No	Epilepsy	Single gene
419	Male	269	Yes	Epilepsy	Single gene
420	Male	3	Yes	NDD	NGS-based
421	Male	141	Yes	NDD	Single gene
422	Female	19	No	NDD	Single gene
423	Female	37	Yes	Epilepsy	Single gene
424	Male	79	No	NDD	Single gene
425	Male	60	Yes	Epilepsy	Single gene
426	Female	126	No	NDD	Single gene
427	Male	34	Yes	Epilepsy	Single gene
428	Male	47	No	NDD	Single gene
429	Female	78	Yes	NDD	NGS-based
430	Female	92	Yes	Epilepsy	Single gene
431	Female	127	Yes	Epilepsy	Single gene
432	Female	51	Yes	Epilepsy	Single gene
433	Female	27	Yes	NDD	Single gene
434	Male	39	Yes	NDD	Single gene
435	Male	146	Yes	NDD	NGS-based
436	Male	67	No	NDD	Single gene
437	Female	21	No	NDD	Single gene

438	Female	13	No	NDD	Single gene
439	Female	6	Yes	NDD	Single gene
440	Male	223	No	NDD	Single gene
441	Female	10	Yes	Epilepsy	Single gene
442	Male	122	Yes	NDD	Single gene
443	Male	18	Yes	Epilepsy	Single gene
444	Male	3	Yes	NDD	Single gene
445	Female	32	Yes	Epilepsy	Single gene
446	Female	64	Yes	NDD	NGS-based
447	Female	3	Yes	NDD	Single gene
448	Female	7	Yes	Epilepsy	Single gene
449	Female	25	No	NDD	Single gene
450	Male	34	No	NDD	Single gene
451	Male	42	Yes	Epilepsy	Single gene
452	Male	10	Yes	Epilepsy	Single gene
453	Female	2	Yes	Epilepsy	Single gene
454	Female	2	Yes	Epilepsy	Single gene
455	Male	14	Yes	Epilepsy	Single gene
456	Male	3	Yes	Epilepsy	Single gene
457	Male	4	Yes	Epilepsy	Single gene
458	Male	10	Yes	NDD	Single gene
459	Male	66	Yes	Epilepsy	Single gene
460	Male	71	No	NDD	Single gene
461	Female	23	Yes	Epilepsy	Single gene
462	Male	4	Yes	Epilepsy	Single gene
463	Female	27	Yes	Epilepsy	Single gene
464	Female	131	Yes	Epilepsy	Single gene
465	Male	21	No	NDD	Single gene
466	Male	82	Yes	NDD	Single gene
467	Female	271	Yes	NDD	NGS-based
468	Female	45	No	NDD	Single gene

469	Male	45	Yes	NDD	Single gene
470	Female	46	No	MCD	Single gene
471	Male	55	No	NDD	Single gene
472	Male	211	Yes	Epilepsy	Single gene
473	Male	21	No	NDD	Single gene
474	Male	65	Yes	NDD	Single gene
475	Male	71	Yes	MCD	Single gene
476	Female	5	Yes	MCD	Single gene
477	Female	24	Yes	NDD	Single gene
478	Male	26	Yes	NDD	Single gene
479	Male	26	No	NDD	Single gene
480	Female	140	Yes	NDD	Single gene
481	Male	8	Yes	MCD	Single gene
482	Male	3	No	MCD	Single gene
483	Male	7	Yes	MCD	Single gene
484	Male	48	Yes	NDD	Single gene
485	Male	23	No	MCD	Single gene
486	Male	1	No	MCD	Single gene
487	Male	65	Yes	NDD	Single gene
488	Male	5	Yes	Epilepsy	Single gene
489	Male	53	Yes	Epilepsy	Single gene
490	Male	58	No	NDD	Single gene
491	Male	45	Yes	NDD	Single gene
492	Male	49	Yes	Epilepsy	Single gene
493	Male	192	Yes	NDD	Single gene
494	Male	15	Yes	Epilepsy	Single gene
495	Male	42	Yes	NDD	Single gene
496	Female	8	Yes	Epilepsy	Single gene
497	Male	40	No	NDD	NGS-based
498	Male	142	Yes	NDD	NGS-based

499	Male	132	Yes	NDD	NGS-based
500	Female	53	Yes	NDD	NGS-based
501	Male	110	Yes	NDD	NGS-based
502	Male	2	No	NDD	NGS-based
503	Male	97	No	NDD	NGS-based
504	Male	31	Yes	NDD	NGS-based
505	Male	27	Yes	NDD	NGS-based
506	Male	153	Yes	NDD	NGS-based
507	Male	65	Yes	NDD	NGS-based
508	Male	44	Yes	NDD	NGS-based
509	Male	58	Yes	NDD	NGS-based
510	Female	71	Yes	NDD	NGS-based
511	Male	8	Yes	Epilepsy	NGS-based
512	Male	103	Yes	NDD	NGS-based
513	Female	26	No	NDD	NGS-based
514	Female	94	Yes	NDD	NGS-based
515	Male	46	Yes	NDD	NGS-based
516	Male	55	No	NDD	NGS-based
517	Male	45	No	NDD	NGS-based
518	Female	1	Yes	NDD	NGS-based
519	Female	222	Yes	NDD	NGS-based
520	Male	67	No	NDD	NGS-based
521	Male	23	Yes	NDD	NGS-based
522	Female	20	Yes	NDD	NGS-based
523	Male	72	Yes	NDD	NGS-based
524	Male	144	Yes	NDD	NGS-based
525	Female	17	Yes	Epilepsy	NGS-based
526	Female	16	Yes	NDD	NGS-based
527	Female	56	No	NDD	NGS-based
528	Male	29	Yes	NDD	NGS-based
529	Female	47	Yes	NDD	NGS-based
530	Male	132	Yes	NDD	NGS-based

531	Female	28	Yes	Epilepsy	NGS-based
532	Male	9	Yes	NDD	NGS-based
533	Male	6	Yes	Epilepsy	NGS-based
534	Female	53	Yes	Epilepsy	NGS-based
535	Male	60	Yes	NDD	NGS-based
536	Female	6	Yes	NDD	NGS-based
537	Male	29	Yes	NDD	NGS-based
538	Male	83	No	NDD	NGS-based
539	Male	10	No	NDD	NGS-based
540	Male	34	Yes	Epilepsy	NGS-based
541	Female	157	Yes	Epilepsy	NGS-based
542	Female	276	No	NDD	NGS-based
543	Male	34	Yes	NDD	NGS-based
544	Male	48	No	NDD	NGS-based
545	Female	36	Yes	Epilepsy	NGS-based
546	Female	4	Yes	NDD	NGS-based
547	Male	159	Yes	NDD	NGS-based
548	Male	125	Yes	NDD	NGS-based
549	Male	45	Yes	Epilepsy	NGS-based
550	Female	13	No	NDD	NGS-based
551	Male	98	No	NDD	NGS-based
552	Female	72	Yes	NDD	NGS-based
553	Female	45	No	NDD	NGS-based
554	Male	16	Yes	NDD	NGS-based
555	Female	62	Yes	Epilepsy	NGS-based
556	Female	222	Yes	NDD	NGS-based
557	Male	68	Yes	NDD	NGS-based
558	Female	48	No	NDD	NGS-based
559	Female	115	No	NDD	NGS-based
560	Male	66	Yes	NDD	NGS-based
561	Female	31	No	NDD	NGS-based
562	Female	52	No	NDD	NGS-based

563	Male	25	No	NDD	NGS-based

NDD, neurodevelopment disorders. CES, clinical exome sequencing.

Supplementary Table 3. Comparison of time for trio test, diagnostic yield, and cases solved by the trio test between the initial trio and delayed trio.

	Delayed trio (n = 526)	Initial trio (n = 37)
The time interval†	2.2 (IQR, 1.4-3.5)	0 (IQR, 0-0.2)
Diagnostic yield of trio test	28.9% (152/526)	45.9% (17/37)
Cases solved by trio test	20.0% (105/526)	18.9% (7/37)
Unsolved *de novo* variants which need further investigation	1.3% (7/526)	24.3% (9/37)

IQR, interquartile range.

†The time interval: initial test report to final test report

Supplementary Table 4. *De novo* variants with uncertain significance detected in this study.

Trio#	NDD with epilepsy	Gene	Transcript	Nucleotide	AminoAcid
2	Yes	*LAMA3*	NM_198129.1	c.4171C>T	p.Arg1391Ter
9	Yes	19q13.2 deletion			
52	No	*ATP8B1*	NM_005603.4	c.2285+1G>A	
61	No	*DLX6*	NM_005222.3	c.224A>G	p.His75Arg
108	Yes	*COPA*	NM_004371.3	c.2476+1G>T	
113	Yes	*CUX1*	NM_001913.4	c.1560G>C	p.Glu520Asp
130	Yes	*DNM2*	NM_001005360.2	c.198dup	p.Arg67AlafsTer29
157	No	*GAL3ST2*	NM_022134.2	c.936_937dup	p.Arg313ProfsTer59
277	Yes	*CTC1*	NM_025099.5	c.1077+1G>C	
291	Yes	*LDHA*	NM_005566.3	c.759_778del	p.Leu254ArgfsTer8
324	No	*GATA3*	NM_001002295.1	c.307C>G	p.Leu103Val
327	Yes	*RABL6*	NM_024718.4	c.1874C>G	p.Ser625Trp
330	Yes	*RANBP17*	NM_022897.3	c.1668T>C	p.Phe556=
333	Yes	*RBM12*	NM_001198838.1	c.1900del	p.Gln634LysfsTer6
407	Yes	*VPS13A*	NM_033305.2	c.7657G>C	p.Asn2553His
467	Yes	*TDRD7*	NM_014290.2	c.3037C>T	p.Arg1013Ter

NDD, neurodevelopment disorders.
